# Supplementary material for: Toolbox Accelerating Glycomics (TAG): Glycan Annotation from MALDI-TOF MS Spectra and Mapping Expression Variation to Biosynthetic Pathways
Source: Biomolecules. 2020 Sep 28;10(10):1383. doi: 10.3390/biom10101383 (PMC7650810; doi:10.3390/biom10101383)
Supplement: Supplementary file 1 [file biomolecules-10-01383-s001.zip › biomolecules-939710-figures proofreading.pdf]

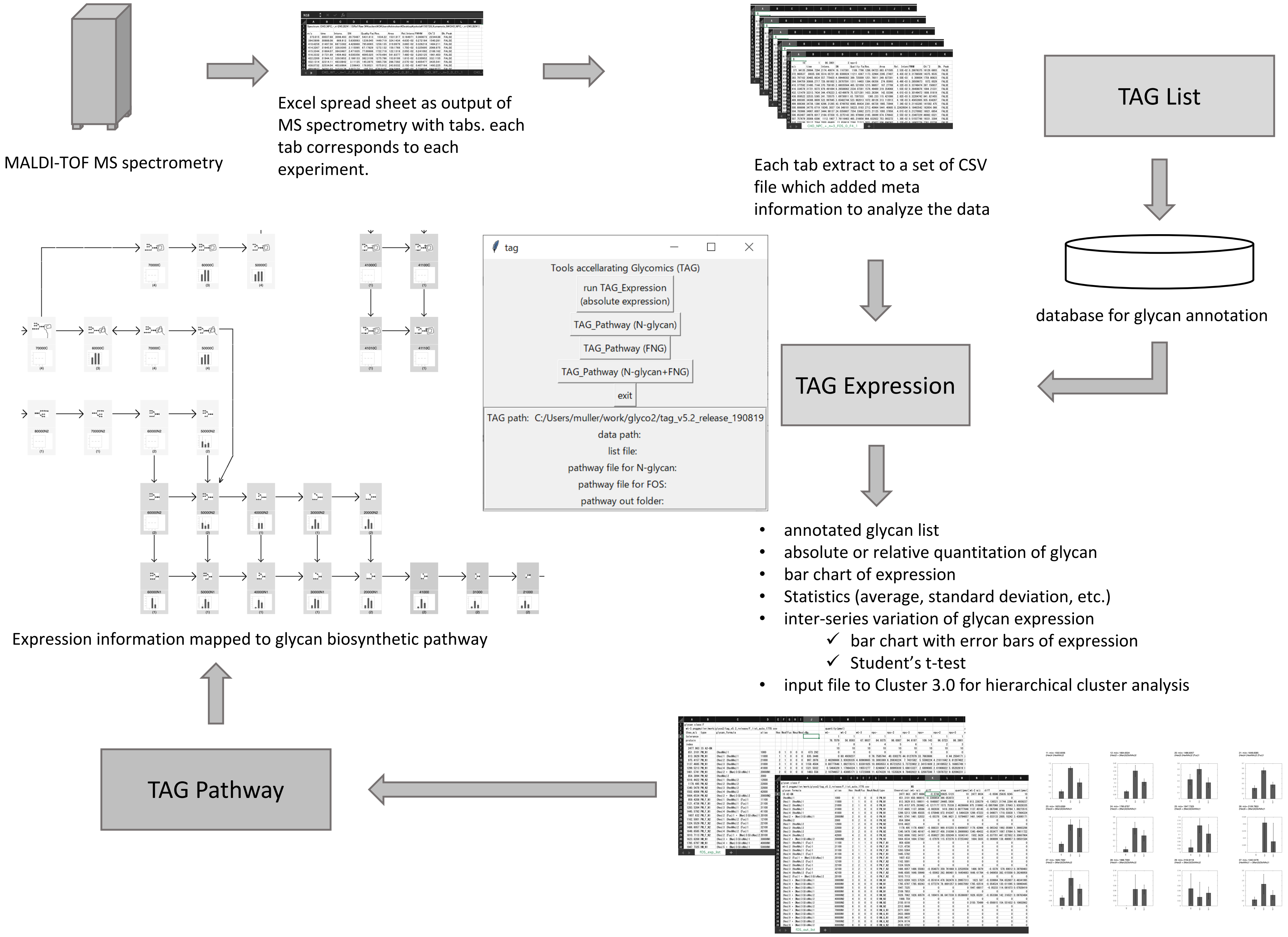
MALDI-TOF MS spectrometry
Excel spread sheet as output of MS spectrometry with tabs. each tab corresponds to each experiment.
Each tab extract to a set of CSV file which added meta information to analyze the data
TAG List
database for glycan annotation
TAG Expression
tag
Tools accellarating Glycomics (TAG)
run TAG_Expression (absolute expression)
TAG_Pathway (N-glycan)
TAG_Pathway (FNG)
TAG_Pathway (N-glycan+FNG)
exit
TAG path: C:/Users/muller/work/glyco2/tag_v5.2_release_190819
data path:
list file:
pathway file for N-glycan:
pathway file for FOS:
pathway out folder:
annotated glycan list
absolute or relative quantitation of glycan
bar chart of expression
Statistics (average, standard deviation, etc.)
inter-series variation of glycan expression
✓ bar chart with error bars of expression
✓ Student's t-test
input file to Cluster 3.0 for hierarchical cluster analysis
Expression information mapped to glycan biosynthetic pathway
TAG Pathway

	A	B	C
1	N		
2	IS A2GN	2477.963	
3	(Hex) 1 (HexNAc) 2	1016.4422	PM
4	(Hex) 2 (HexNAc) 2	1178.495	PM
5	(Hex) 3 (HexNAc) 2	1340.5478	PM
6	(Hex) 4 (HexNAc) 2	1502.6006	PM
7	(Hex) 2 + (Man) 3 (GlcNAc) 2	1664.6534	HM
8	(Hex) 1 (HexNAc) 2 (Fuc) 1	1162.5001	PM_F
9	(Hex) 2 (HexNAc) 2 (Fuc) 1	1324.5529	PM_F
10	(Hex) 3 (HexNAc) 2 (Fuc) 1	1486.6057	PM_F
11	(Hex) 4 (HexNAc) 2 (Fuc) 1	1648.6585	PM_F
12	(Hex) 2 (Fuc) 1 + (Man) 3 (GlcNAc) 2	1810.7113	HM_F
13	(Hex) 3 + (Man) 3 (GlcNAc) 2	1826.7062	HM
14	(Hex) 4 + (Man) 3 (GlcNAc) 2	1988.759	HM
15	(Hex) 5 + (Man) 3 (GlcNAc) 2	2150.8118	HM
16	(Hex) 6 + (Man) 3 (GlcNAc) 2	2312.8646	HM
17	(Hex) 7 + (Man) 3 (GlcNAc) 2	2474.9174	HM_G
18	(Hex) 8 + (Man) 3 (GlcNAc) 2	2636.9702	HM_G
19	(Hex) 9 + (Man) 3 (GlcNAc) 2	2799.023	HM_G
20	(HexNAc) 1 + (Man) 3 (GlcNAc) 2	1543.6271	1_n

	A	B	C
1	F		
2	IS A2GN	2477.963	
3	(HexNAc) 1	651.3101	PM_N1
4	(Hex) 1 (HexNAc) 1	813.3629	PM_N1
5	(Hex) 2 (HexNAc) 1	975.4157	PM_N1
6	(Hex) 3 (HexNAc) 1	1137.4685	PM_N1
7	(Hex) 4 (HexNAc) 1	1299.5213	PM_N1
8	(Hex) 2 + (Man) 3 (GlcNAc) 1	1461.5741	HM_N1
9	(HexNAc) 2	854.3894	PM_N2
10	(Hex) 1 (HexNAc) 2	1016.4422	PM_N2
11	(Hex) 2 (HexNAc) 2	1178.495	PM_N2
12	(Hex) 3 (HexNAc) 2	1340.5478	PM_N2
13	(Hex) 4 (HexNAc) 2	1502.6006	PM_N2
14	(Hex) 2 + (Man) 3 (GlcNAc) 2	1664.6534	HM_N2
15	(Hex) 1 (HexNAc) 1 (Fuc) 1	959.4208	PM_F_N1
16	(Hex) 2 (HexNAc) 1 (Fuc) 1	1121.4736	PM_F_N1
17	(Hex) 3 (HexNAc) 1 (Fuc) 1	1283.5264	PM_F_N1
18	(Hex) 4 (HexNAc) 1 (Fuc) 1	1445.5792	PM_F_N1
19	(Hex) 2 (Fuc) 1 + (Man) 3 (GlcNAc) 1	1607.632	HM_F_N1
20	(Hex) 1 (HexNAc) 2 (Fuc) 1	1162.5001	PM_F_N2

	A	B	C	D	E	F	G	H	I	J	K	L
1	Spectrum: CHO_WT_-_n=1_2¥0_A5¥1 1SRef Raw (¥¥tsclient¥C¥Users¥shinoken¥Desktop¥yokota¥150728_Kumamoto_N¥CHO_WT_-_n=1_2¥0_A5¥1)											
2	5	2	17.0462	1	wt-							
3	m/z	time	Intens.	SN	Quality Fac	Res.	Area	Rel. Intens	FWHM	Chi^2	Bk. Peak	
4	371.933902	29994.4312	1057.02446	9.27755578	901.318813	1354.78275	405.043875	5.52E-02	0.27453398	5074.75827	FALSE	
5	372.982979	30036.4032	2835.02979	24.8708036	6241.39797	1216.13387	1164.95528	0.14803197	0.30669566	5314.89986	FALSE	
6	394.600625	30888.4997	299.181037	2.57081923	299.967749	1440.81111	112.571559	1.56E-02	0.27387395	366.967776	FALSE	
7	416.322194	31721.4686	277.467115	2.36286978	327.523771	1611.67872	100.802826	1.45E-02	0.25831587	251.538758	FALSE	
8	460.741688	33359.6749	225.452088	1.91246743	72.3547795	936.852364	165.308706	1.18E-02	0.49179754	1130.0737	FALSE	
9	489.793767	34388.63	310.761287	2.64422963	192.092042	971.050996	209.677113	1.62E-02	0.50439552	683.473098	FALSE	
10	499.789887	34735.5696	3176.69793	27.0279262	2041.75011	2111.73877	1136.35632	0.16587228	0.23667221	85595.0972	FALSE	
11	500.773421	34769.517	10116.6128	86.0650008	19915.4782	2455.84702	3197.13522	0.5282421	0.20391067	105438.148	FALSE	
12	503.003213	34846.3568	2165.55108	18.4239271	250.215585	1991.42365	718.312988	0.11307493	0.25258473	68322.4512	FALSE	
13	503.97475	34879.783	6239.2702	52.9718087	2981.18338	2451.24087	1738.71648	0.32578544	0.20559985	49965.9673	FALSE	
14	504.771905	34907.1856	2151.39822	18.2967291	1122.92348	2360.26603	655.477557	0.11233593	0.21386229	16352.2451	FALSE	
15	505.895025	34945.7565	401.850572	3.41974498	92.5747716	1181.94944	225.88703	2.10E-02	0.42801748	7222.5017	FALSE	
16	506.797358	34976.714	3667.92051	31.1975458	5660.674	2203.85002	1160.5866	0.19152162	0.22996	7980.41949	FALSE	
17	507.727927	35008.6113	862.634885	7.35358755	1095.16542	956.056743	637.91536	4.50E-02	0.53106464	4419.51936	FALSE	
18	508.769903	35044.2927	416.548457	3.53618923	303.926967	2162.77263	155.290449	2.18E-02	0.23523966	1589.01111	FALSE	
19	509.788473	35079.1371	302.392296	2.57542386	64.5081078	1979.70745	112.533143	0.01578951	0.25750697	1901.78933	FALSE	
20	510.758895	35112.3021	730.958313	6.2097764	623.15645	2377.25653	241.7814	3.82E-02	0.21485224	2195.08722	FALSE	
21	511.757616	35146.4013	1015.05373	8.63497846	926.845481	2861.32574	279.794123	5.30E-02	0.17885332	2585.41625	FALSE	
22	512.845201	35183.4967	518.486619	4.41513554	208.530672	1918.12289	194.179237	2.71E-02	0.26736827	3303.65928	FALSE	
23	516.029872	35291.8938	7827.20523	66.666402	11814.1571	2708.37276	2054.9631	0.40869997	0.19053133	31690.1249	FALSE	
24	517.989859	35358.4397	1757.78865	14.9710105	322.745419	2928.50692	432.346579	9.18E-02	0.17687848	34410.5263	FALSE	

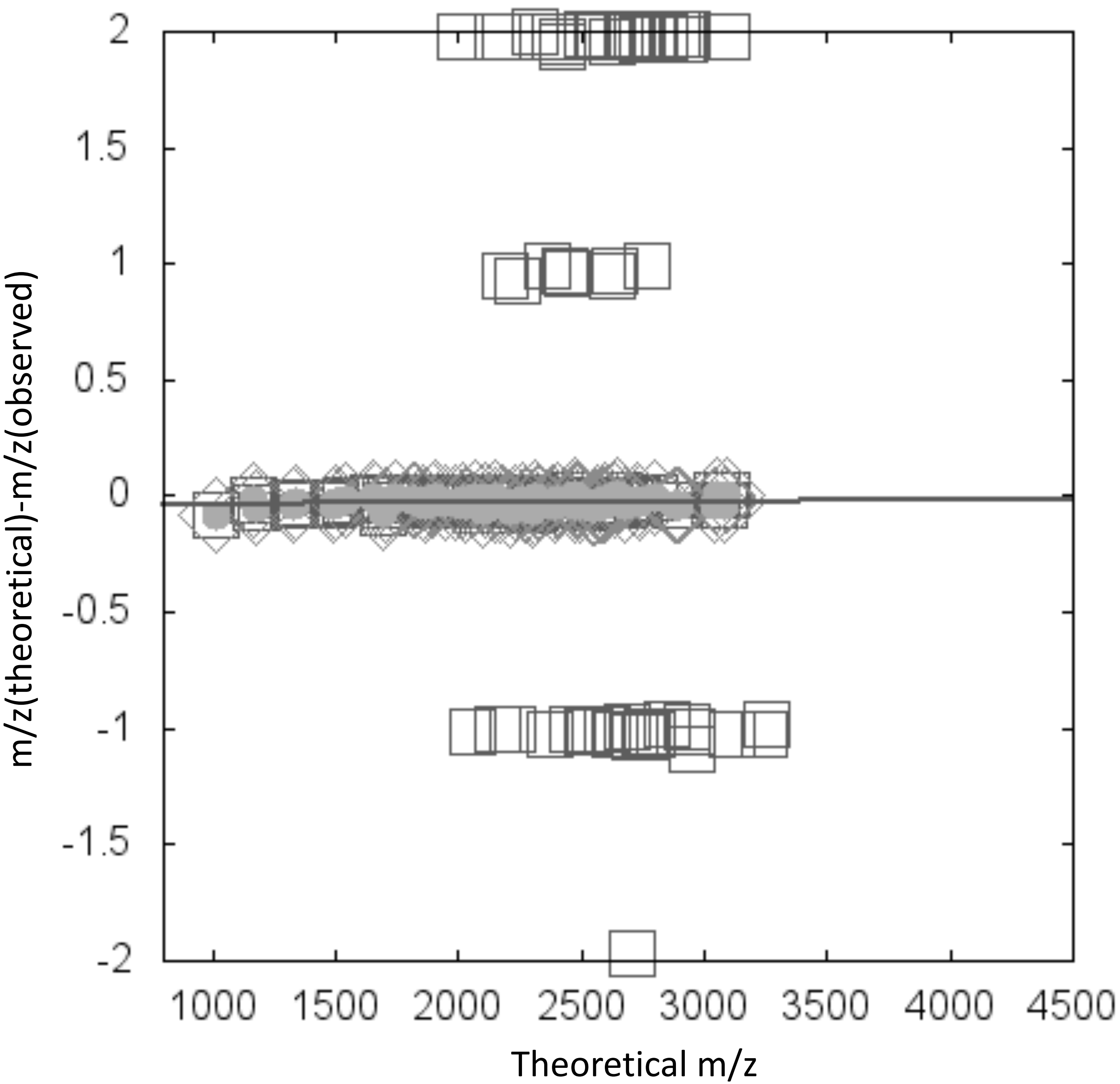

m/z(theoretical)-m/z(observed)
2
1.5
1
0.5
0
-0.5
-1
-1.5
-2
1000
1500
2000
2500
3000
3500
4000
4500
Theoretical m/z

	BR	BS	BT	BU	BV	BW
38	./glyco_img/ra.png		./glyco_img/ra.png		./glyco_img/ra.png	
39		N-glycan_folded		N-glycan_folded		N-glycan_folded
40		HM_GN2		HM_GN2		HM_GN2
41		50000C		40000C		30000C
42		Golgi and later		Golgi and later		Golgi and later
43				./glyco_img/da.png		
44						

	A	B	C	D	E	F	G	H	I
1	glycan class:N								
2	wt-3.pnggmuller/work/glyco2/tag_v5.2_release/N_list_auto_877.csv								
3	glycan formula	alias	Hex	HexNAc	Fuc	NeuAc	NeuGc	type	theoretical m/z
4	IS A2-GN								2477.963
5	(Hex)1 (HexNAc)2	12000	1	2	0	0	0	PM	1016.4422
6	(Hex)2 (HexNAc)2	22000	2	2	0	0	0	PM	1178.495
7	(Hex)3 (HexNAc)2	32000	3	2	0	0	0	PM	1340.5478
8	(Hex)4 (HexNAc)2	42000	4	2	0	0	0	PM	1502.6006
9	(Hex)2 + (Man)3(GlcNAc)2	20000C	2	0	0	0	0	HM	1664.6534
10	(Hex)1 (HexNAc)2 (Fuc)1	12100	1	2	1	0	0	PM_F	1162.5001
11	(Hex)2 (HexNAc)2 (Fuc)1	22100	2	2	1	0	0	PM_F	1324.5529
12	(Hex)3 (HexNAc)2 (Fuc)1	32100	3	2	1	0	0	PM_F	1486.6057

glycan structure

five-digit notation

the number of monosaccharide residue

type of glycan in table S1/S2

theoretical m/z

J	K	L	M	N	O	P	Q
MS							
wt- m/z	diff	area	quant(pmol)	wt-2 m/z	diff	area	quant(pmol)
2477.93	-0.0303	22113.89	5.0000	2477.97	0.0069	15451.19	5.0000
0.00	0.0000	0.00	0.0000	0.00	0.0000	0.00	0.0000
1178.45	-0.0435	1387.11	1.8399	1178.46	-0.0302	895.51	2.2934
1340.52	-0.0251	1820.17	2.4143	1340.54	-0.0121	1087.16	2.7843
1502.58	-0.0179	1325.65	1.7583	1502.60	-0.0033	798.54	2.0451
1664.65	-0.0032	6892.52	9.1423	1664.67	0.0121	3939.24	10.0886
1162.42	-0.0771	108.89	0.1444	1162.46	-0.0359	106.23	0.2721
1324.53	-0.0273	3971.36	5.2676	1324.54	-0.0087	2877.42	7.3692
1486.59	-0.0169	734.42	0.9741	1486.58	-0.0209	405.66	1.0389

anotation information (see text)

anotation information (see text) …

	A	B	C	D	E	F	G	H	I	J
1	glycan class:N									
2	wt-3.pnggmuller/work/glyco2/tag_v5.2_release/N_list_877_L.csv									
3	th m/z	type	glycan formula	alias	Hex	HexNAc	Fuc	NeuAc	NeuGc	m/z(Na adduct)
4	tolerance									
5	protein									
6	index									
7	2477.963	IS A2-GN								
8	1178.495	PM	(Hex)2 (HexNAc)2	22000	2	2	0	0	0	1200.4769
9	1340.5478	PM	(Hex)3 (HexNAc)2	32000	3	2	0	0	0	1362.5297
10	1502.6006	PM	(Hex)4 (HexNAc)2	42000	4	2	0	0	0	1524.5825
11	1664.6534	HM	(Hex)2 + (Man)3(GlcNAc)2	20000C	2	0	0	0	0	1686.6353
12	1162.5001	PM_F	(Hex)1 (HexNAc)2 (Fuc)1	12100	1	2	1	0	0	1184.482
13	1324.5529	PM_F	(Hex)2 (HexNAc)2 (Fuc)1	22100	2	2	1	0	0	1346.5348

theoretical m/z

glycan structure

five-digit notation

the number of monosaccharide residue

m/z of Sodium adducted ion

L	M	N	O	P	Q	R	S	T	U	V	W
quantity(pmol)									average		
wt-	wt-2	wt-3	NPC-	NPC-2	NPC-3	NPC+	NPC+2	NPC+3	wt-	NPC-	NPC+
2.0000	2.0000	2.0000	2.0000	2.0000	2.0000	2.0000	2.0000	2.0000			
17.0462	12.6354	15.1097	18.8750	21.7797	21.0266	24.2540	21.3494	19.1978			
7.0000	8.0000	9.0000	4.0000	5.0000	6.0000	1.0000	2.0000	3.0000			
5.0000	5.0000	5.0000	5.0000	5.0000	5.0000	5.0000	5.0000	5.0000			
1.8399	2.2934	1.7380	0.7809	1.7774	1.1562	0.8082	0.8084	0.7958	1.9571	1.2381	0.8041
2.4143	2.7843	2.4335	2.6549	4.8486	3.4934	3.8115	2.9492	3.0561	2.5440	3.6656	3.2723
1.7583	2.0451	1.9872	2.4716	3.4706	2.7488	2.8371	2.8693	2.4514	1.9302	2.8970	2.7193
9.1423	10.0886	9.6376	14.3126	18.3621	15.3609	18.4086	19.4861	16.3759	9.6228	16.0119	18.0902
0.1444	0.2721	0.0000	0.0000	0.0000	0.0000	0.0000	0.0000	0.0000	0.1388	0.0000	0.0000
5.2676	7.3692	5.6320	3.9402	9.8788	7.1982	6.2105	4.6840	4.8444	6.0896	7.0057	5.2463

expression amount of each experiment

average expression of each series

X	Y	Z	AA	AB	AC	AD	AE	AF	AG	AH	AI
std			cv			t-value			p-value		
wt-	NPC-	NPC+	wt-	NPC-	NPC+	wt- - NPC-	wt- - NPC+	NPC- - NPC+	wt- - NPC-	wt- - NPC+	NPC- - NPC+
0.2957	0.5033	0.0072	15.1081	40.6477	0.8949	2.1334	6.7520	1.4935	0.0998	0.0025	0.2096
0.2083	1.1070	0.4700	8.1869	30.1983	14.3638	1.7247	2.4535	0.5665	0.1597	0.0702	0.6013
0.1516	0.5157	0.2326	7.8557	17.8013	8.5522	3.1152	4.9228	0.5441	0.0357	0.0079	0.6153
0.4733	2.1018	1.5793	4.9188	13.1263	8.7304	5.1365	8.8952	1.3692	0.0068	0.0009	0.2428
0.1361	0.0000	0.0000	98.0427	0.0000	0.0000	1.7666	1.7666	0.0000	0.1520	0.1520	1.0000
1.1230	2.9740	0.8388	18.4420	42.4506	15.9891	0.4991	1.0420	0.9862	0.6439	0.3562	0.3799

standard deviations

CVs

t-values and p-values of t-test between all possible combination

11: m/z= 1502.6006
(Hex)4 (HexNAc)2

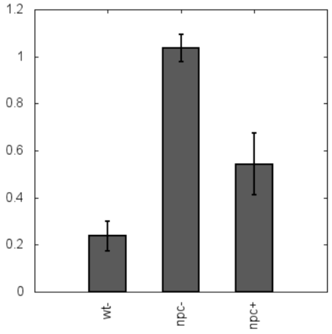

12: m/z= 1664.6534
(Hex)2 + (Man)3(GlcNAc)2

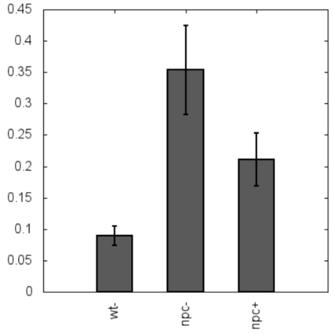

20: m/z= 1486.6057
(Hex)3 (HexNAc)2 (Fuc)1

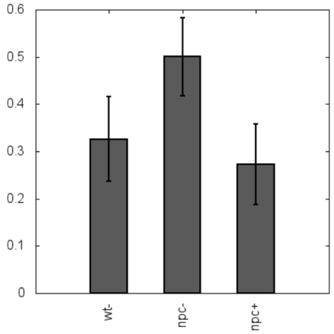

21: m/z= 1648.6585
(Hex)4 (HexNAc)2 (Fuc)1

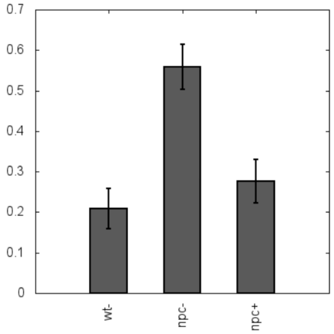

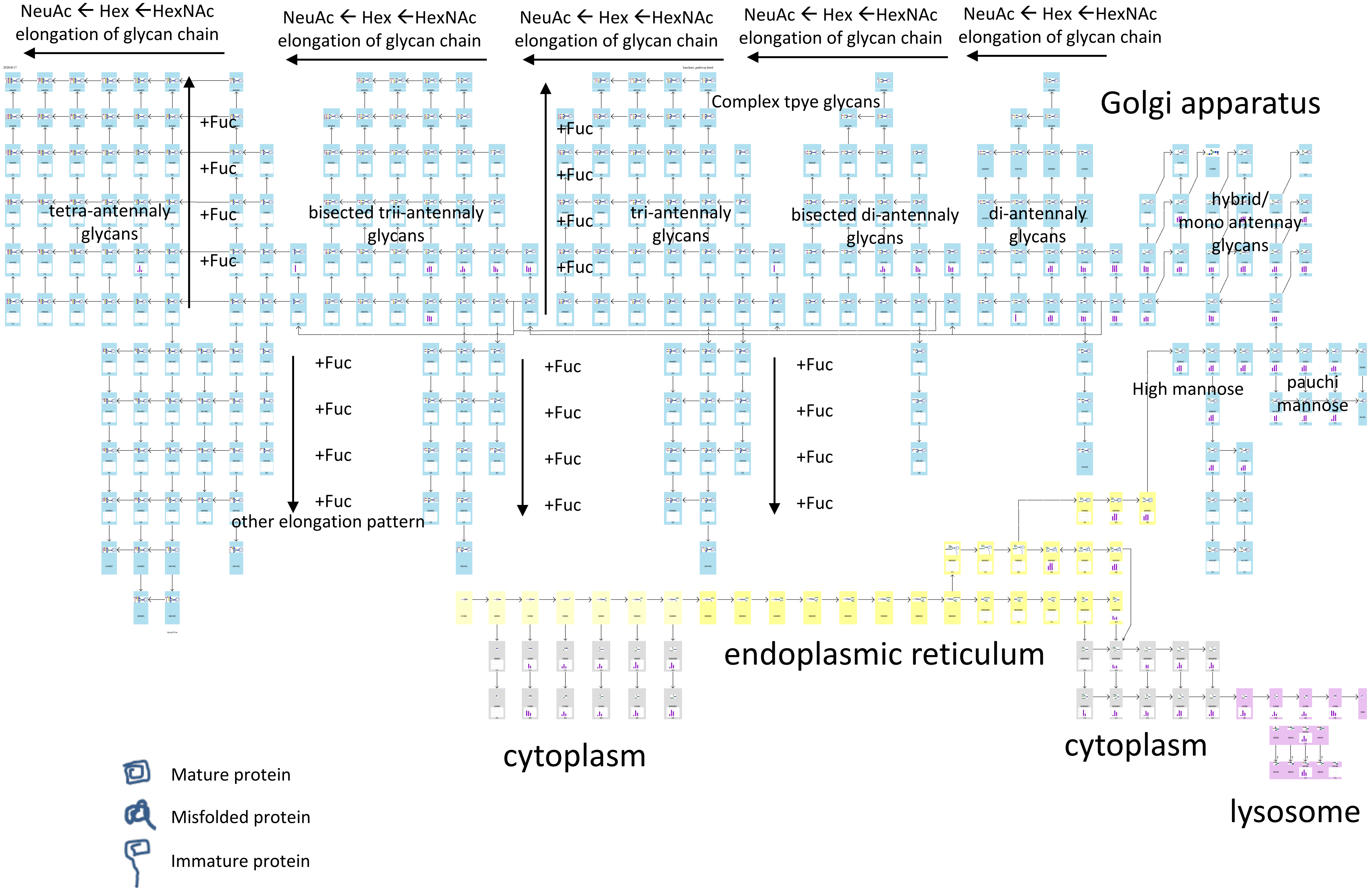
NeuAc ← Hex ←HexNAc
elongation of glycan chain
NeuAc ← Hex ←HexNAc
elongation of glycan chain
NeuAc ← Hex ←HexNAc
elongation of glycan chain
NeuAc ← Hex ←HexNAc
elongation of glycan chain
NeuAc ← Hex ←HexNAc
elongation of glycan chain
Complex tpye glycans
Golgi apparatus
+Fuc
+Fuc
+Fuc
+Fuc
+Fuc
+Fuc
+Fuc
+Fuc
tetra-antennaly glycans
bisected trii-antennaly glycans
tri-antennaly glycans
bisected di-antennaly glycans
di-antennaly glycans
hybrid/ mono antennay glycans
+Fuc
+Fuc
+Fuc
+Fuc
+Fuc
+Fuc
+Fuc
+Fuc
+Fuc
+Fuc
+Fuc
+Fuc
other elongation pattern
High mannose
pauchi mannose
endoplasmic reticulum
cytoplasm
cytoplasm
lysosome
Mature protein
Misfolded protein
Immature protein

Pathway with bar charts

Pathway with expression change and p-values

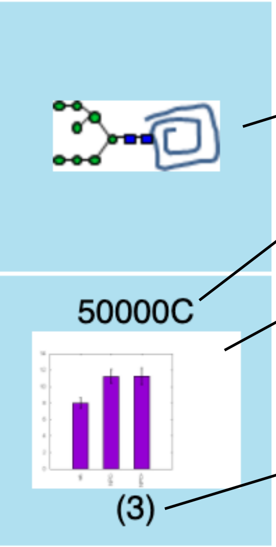

possible structure

five-digit notation

bar chart with error bar

multiplicity: number of candidates on the pathway employed as their m/z are same

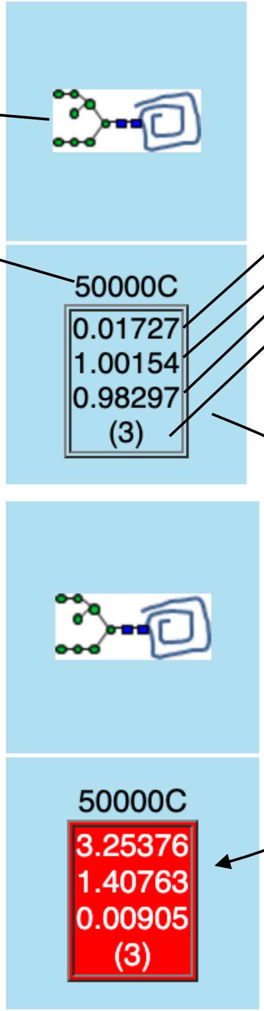

relative abundance

difference

ratio

p-value

multiplicity (number of candidates on the pathway employed as their m/z are same)

Significant increase and decrease are shown in red and deep blue, respectively.

bar chart shown when the glycan is in the list and not detected

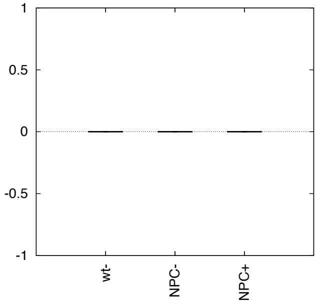

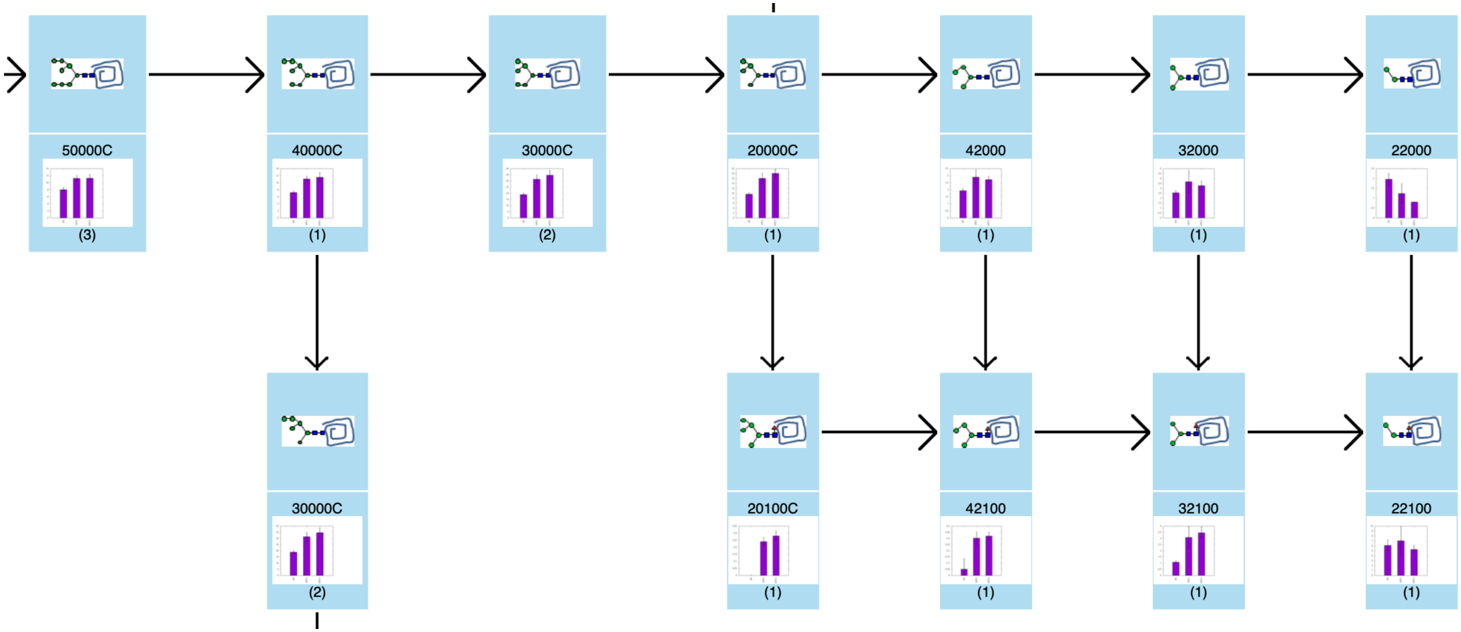
50000C
(3)
40000C
(1)
30000C
(2)
20000C
(1)
42000
(1)
32000
(1)
22000
(1)
30000C
(2)
20100C
(1)
42100
(1)
32100
(1)
22100
(1)

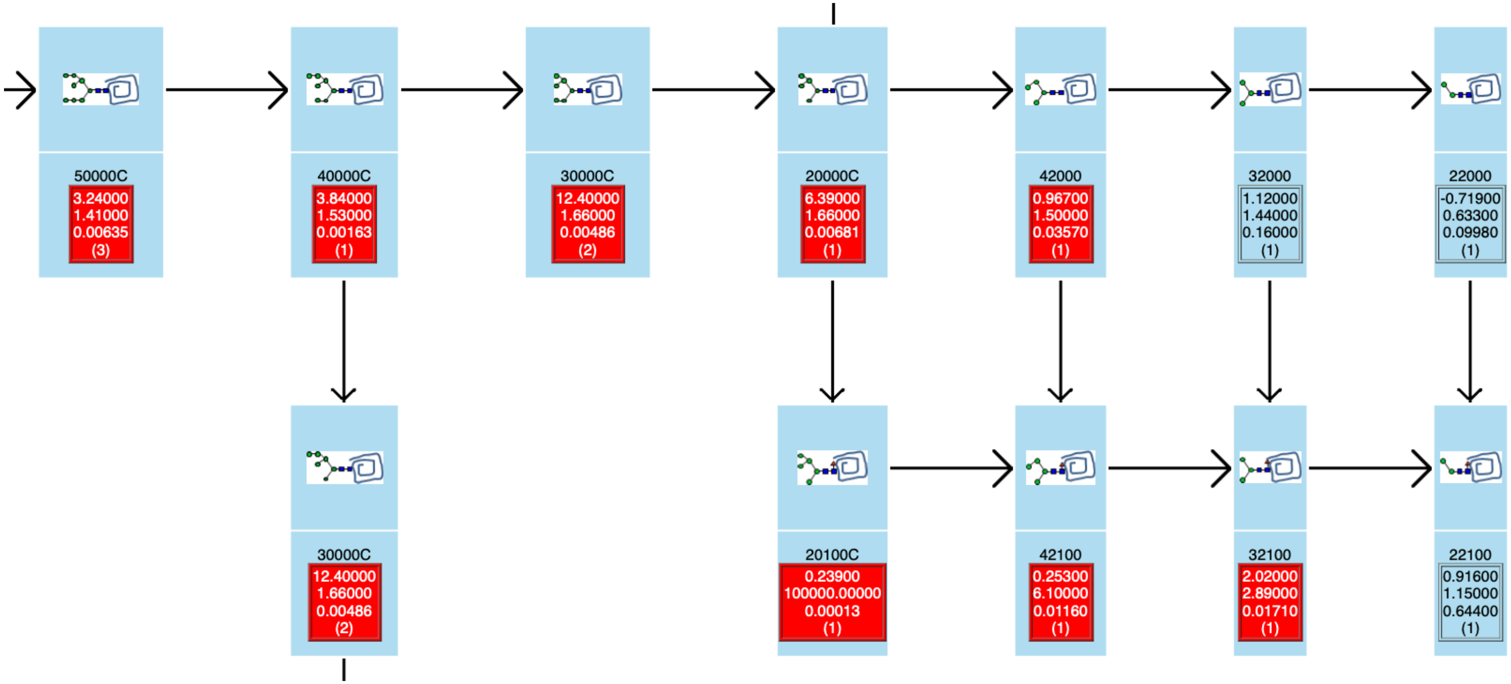
50000C
3.24000
1.41000
0.00635
(3)
40000C
3.84000
1.53000
0.00163
(1)
30000C
12.40000
1.66000
0.00486
(2)
20000C
6.39000
1.66000
0.00681
(1)
42000
0.96700
1.50000
0.03570
(1)
32000
1.12000
1.44000
0.16000
(1)
22000
-0.71900
0.63300
0.09980
(1)
30000C
12.40000
1.66000
0.00486
(2)
20100C
0.23900
100000.00000
0.00013
(1)
42100
0.25300
6.10000
0.01160
(1)
32100
2.02000
2.89000
0.01710
(1)
22100
0.91600
1.15000
0.64400
(1)

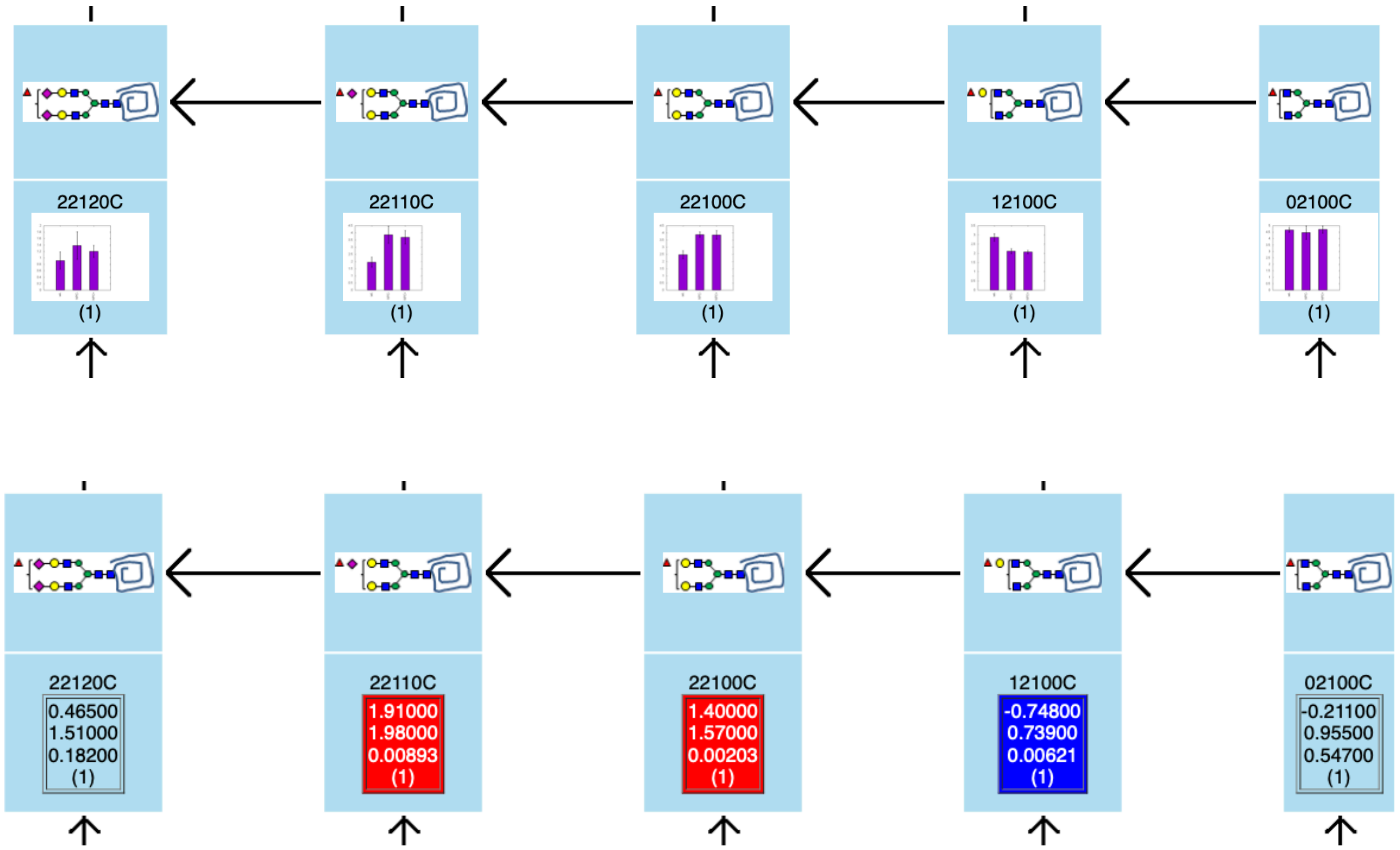
22120C
(1)
22110C
(1)
22100C
(1)
12100C
(1)
02100C
(1)
22120C
0.46500
1.51000
0.18200
(1)
22110C
1.91000
1.98000
0.00893
(1)
22100C
1.40000
1.57000
0.00203
(1)
12100C
-0.74800
0.73900
0.00621
(1)
02100C
-0.21100
0.95500
0.54700
(1)

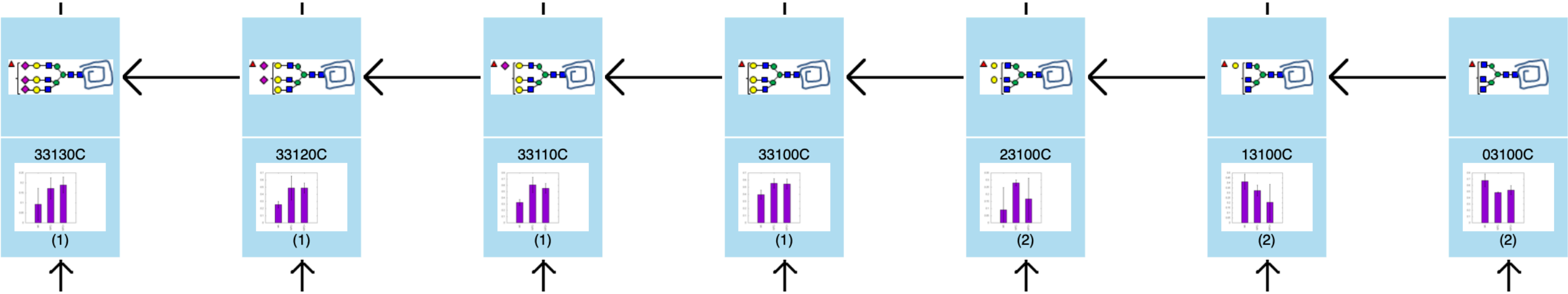
33130C
(1)
33120C
(1)
33110C
(1)
33100C
(1)
23100C
(2)
13100C
(2)
03100C
(2)

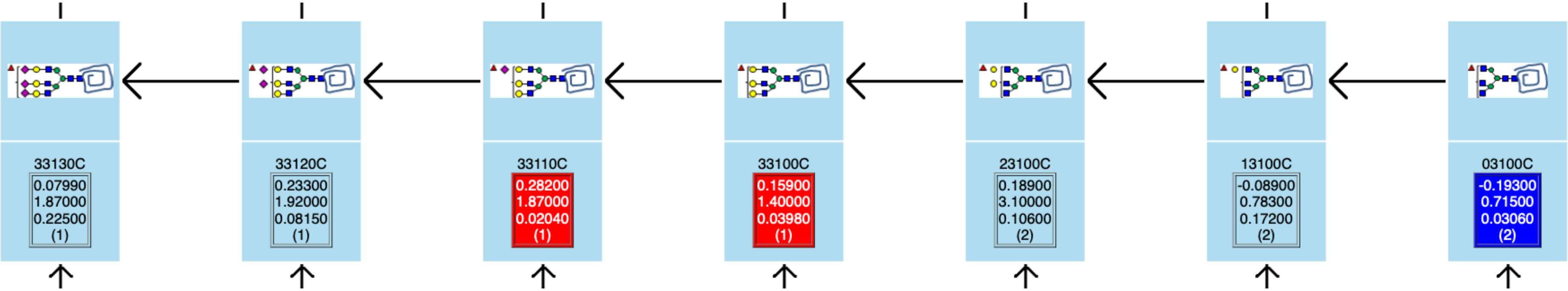
33130C
0.07990
1.87000
0.22500
(1)
33120C
0.23300
1.92000
0.08150
(1)
33110C
0.28200
1.87000
0.02040
(1)
33100C
0.15900
1.40000
0.03980
(1)
23100C
0.18900
3.10000
0.10600
(2)
13100C
-0.08900
0.78300
0.17200
(2)
03100C
-0.19300
0.71500
0.03060
(2)

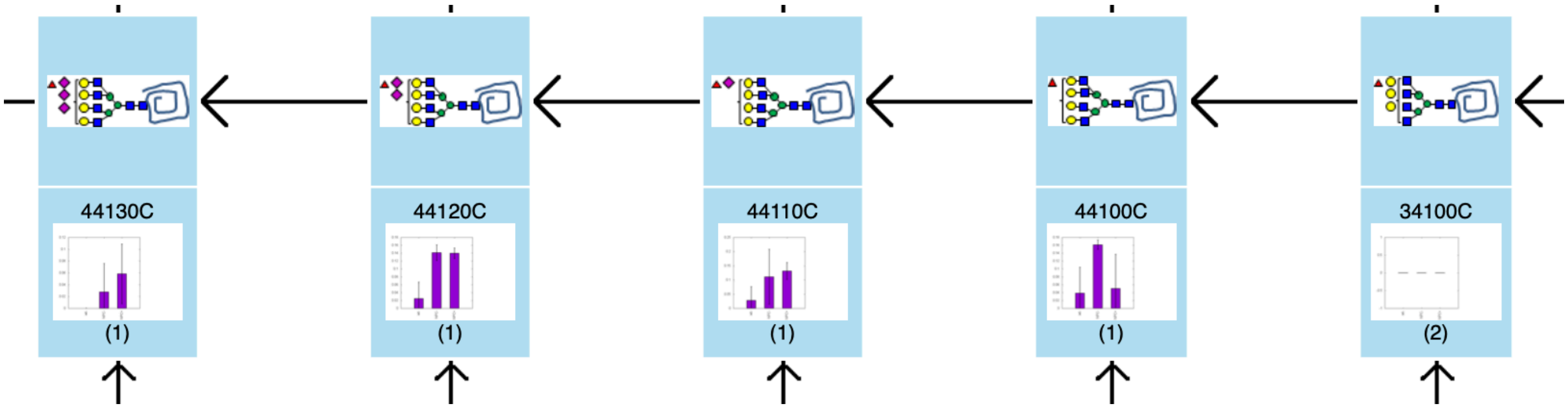
44130C
(1)
44120C
(1)
44110C
(1)
44100C
(1)
34100C
(2)

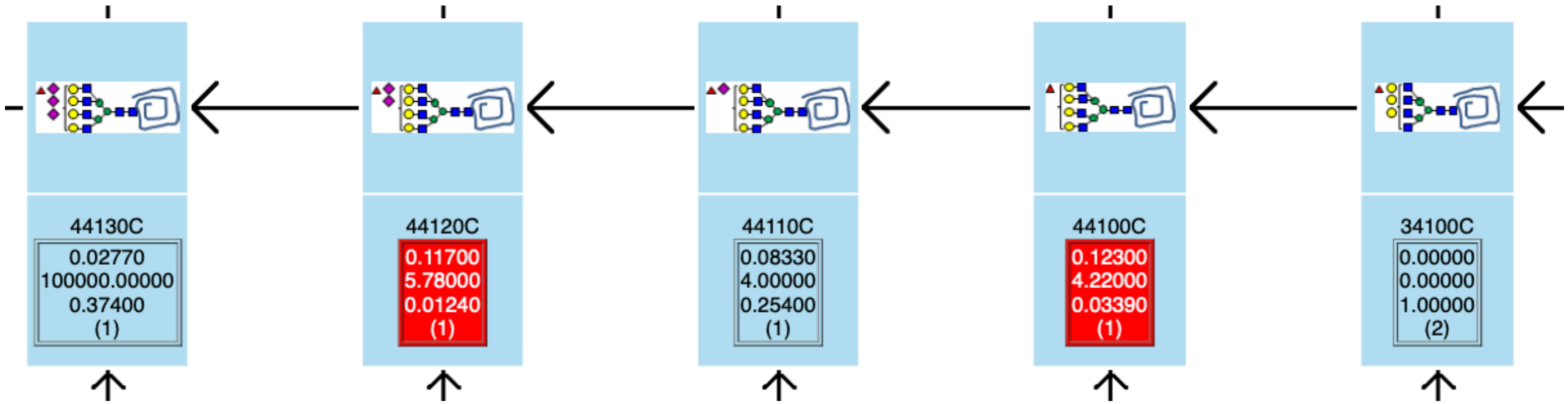
44130C
0.02770
100000.00000
0.37400
(1)
44120C
0.11700
5.78000
0.01240
(1)
44110C
0.08330
4.00000
0.25400
(1)
44100C
0.12300
4.22000
0.03390
(1)
34100C
0.00000
0.00000
1.00000
(2)

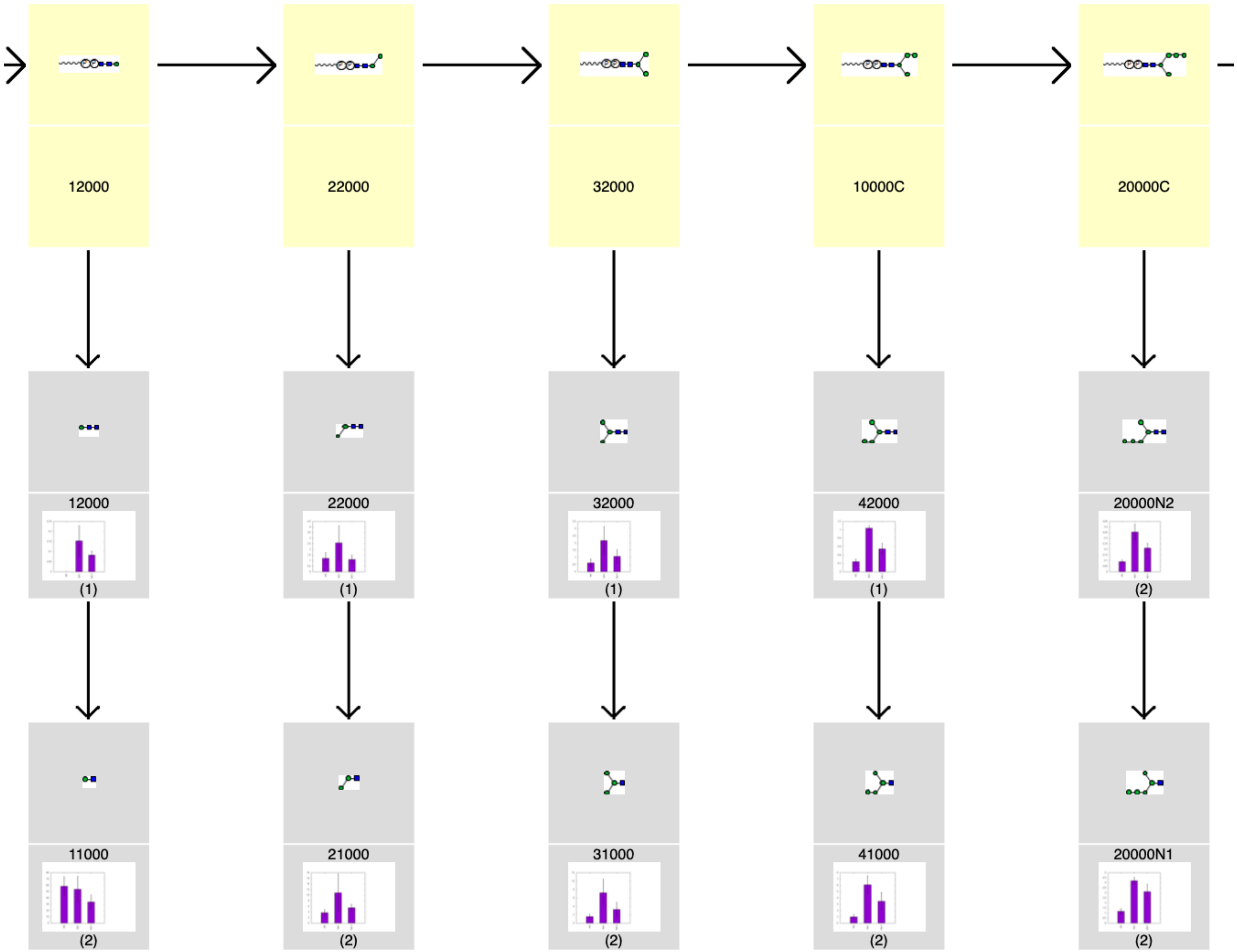
12000
22000
32000
10000C
20000C
12000
22000
32000
42000
20000N2
(1)
(1)
(1)
(1)
(2)
11000
21000
31000
41000
20000N1
(2)
(2)
(2)
(2)
(2)

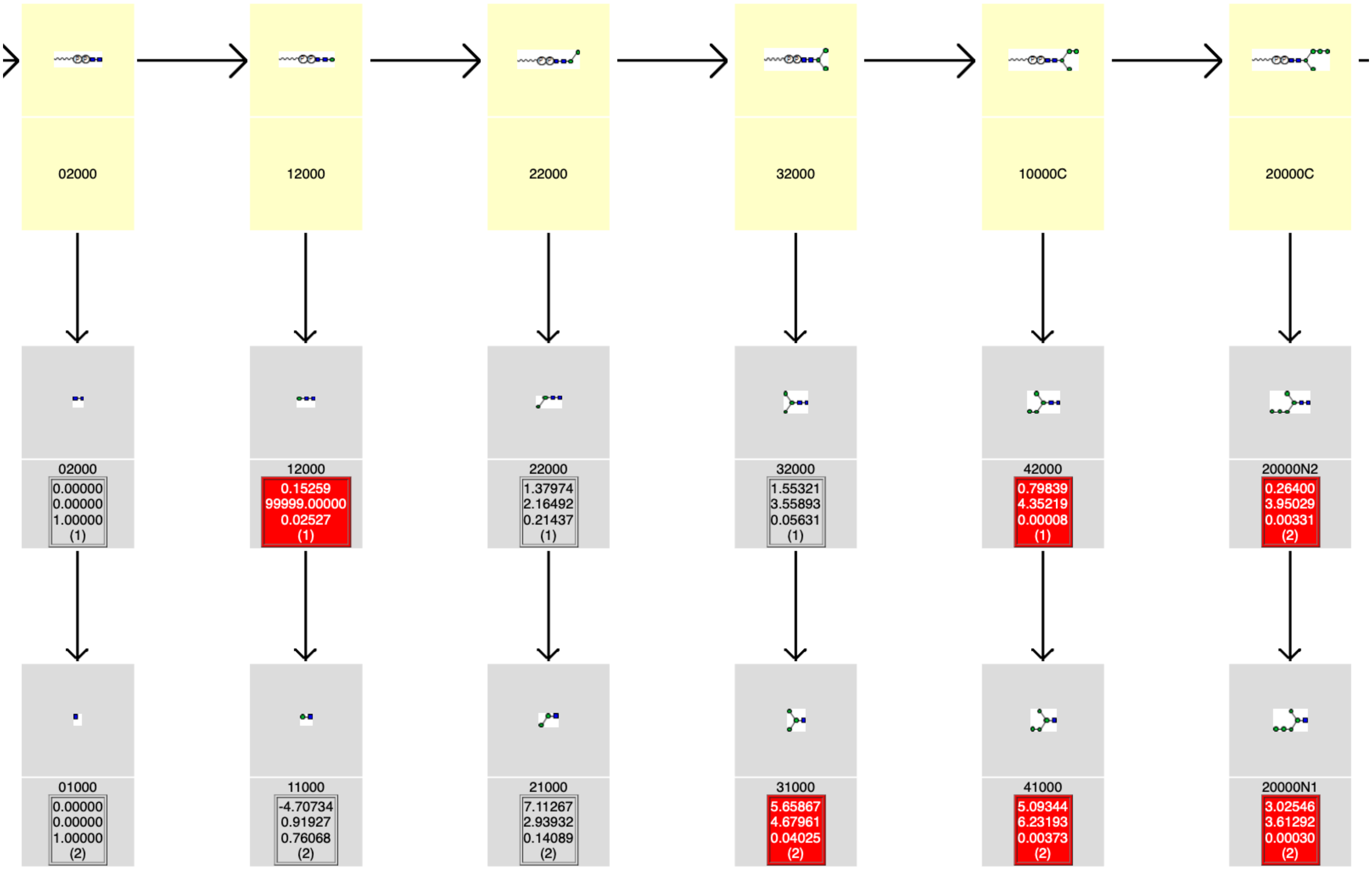

02000
12000
22000
32000
10000C
20000C
02000
0.00000
0.00000
1.00000
(1)
12000
0.15259
99999.00000
0.02527
(1)
22000
1.37974
2.16492
0.21437
(1)
32000
1.55321
3.55893
0.05631
(1)
42000
0.79839
4.35219
0.00008
(1)
20000N2
0.26400
3.95029
0.00331
(2)
01000
0.00000
0.00000
1.00000
(2)
11000
-4.70734
0.91927
0.76068
(2)
21000
7.11267
2.93932
0.14089
(2)
31000
5.65867
4.67961
0.04025
(2)
41000
5.09344
6.23193
0.00373
(2)
20000N1
3.02546
3.61292
0.00030
(2)

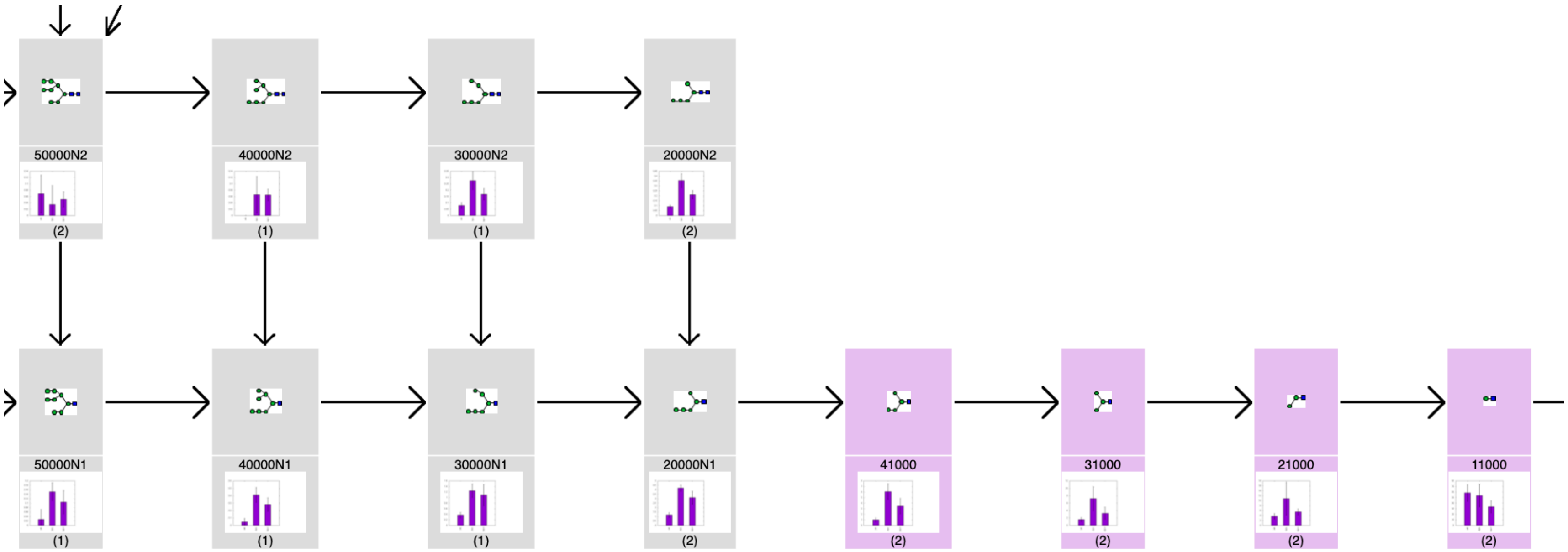
50000N2
(2)
40000N2
(1)
30000N2
(1)
20000N2
(2)
50000N1
(1)
40000N1
(1)
30000N1
(1)
20000N1
(2)
41000
(2)
31000
(2)
21000
(2)
11000
(2)

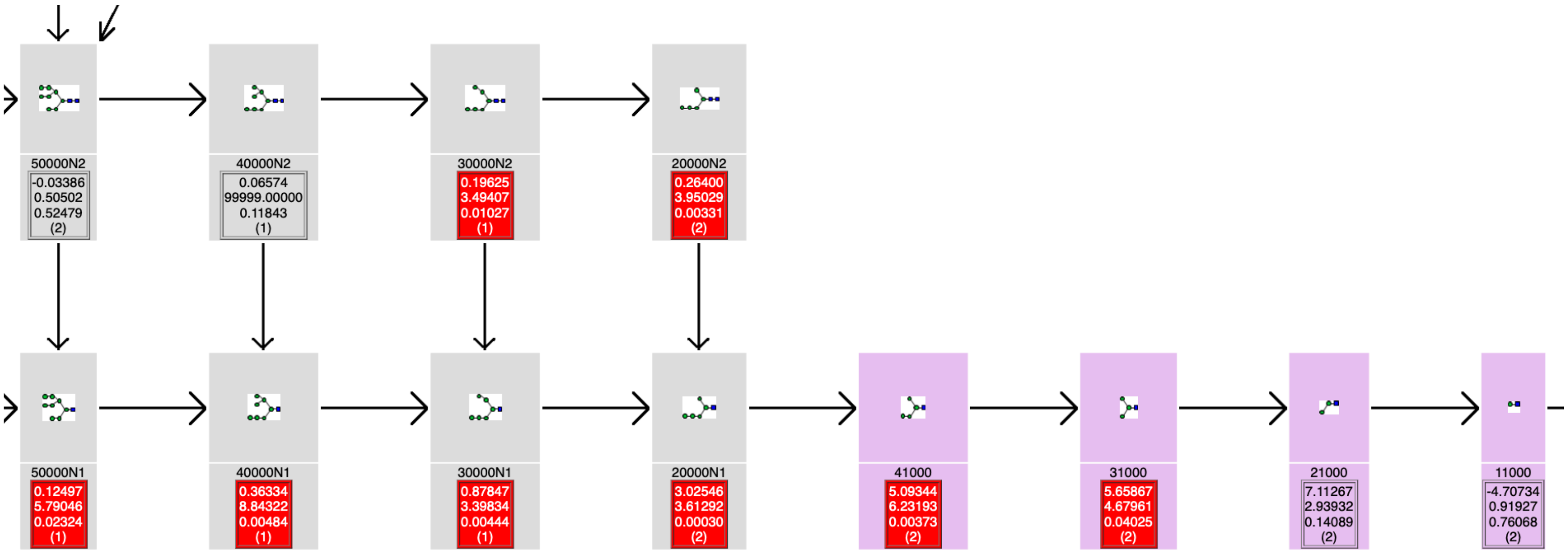
50000N2
-0.03386
0.50502
0.52479
(2)
40000N2
0.06574
99999.00000
0.11843
(1)
30000N2
0.19625
3.49407
0.01027
(1)
20000N2
0.26400
3.95029
0.00331
(2)
50000N1
0.12497
5.79046
0.02324
(1)
40000N1
0.36334
8.84322
0.00484
(1)
30000N1
0.87847
3.39834
0.00444
(1)
20000N1
3.02546
3.61292
0.00030
(2)
41000
5.09344
6.23193
0.00373
(2)
31000
5.65867
4.67961
0.04025
(2)
21000
7.11267
2.93932
0.14089
(2)
11000
-4.70734
0.91927
0.76068
(2)

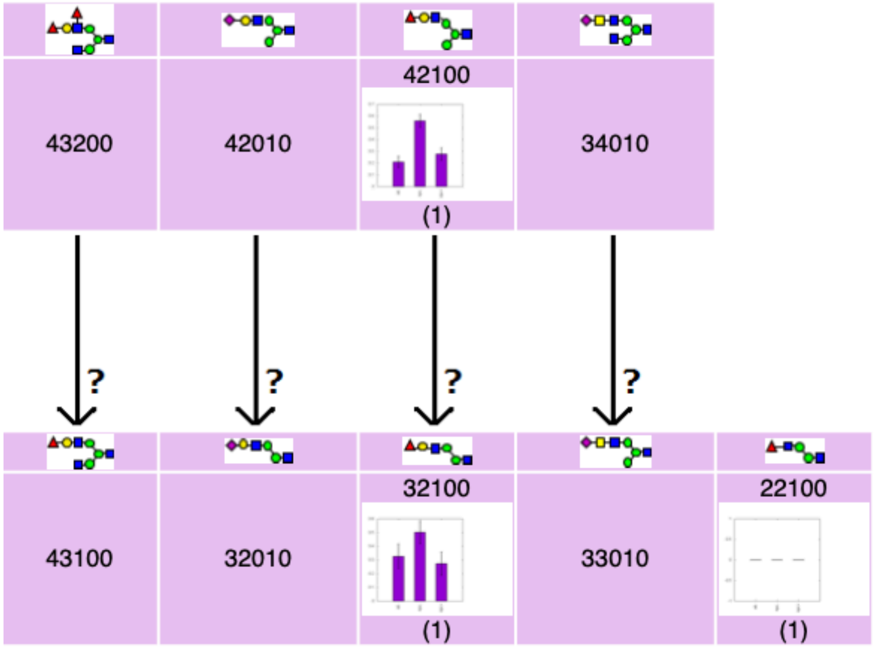
43200
42010
42100
(1)
34010
?
?
?
?
43100
32010
32100
(1)
33010
22100
(1)

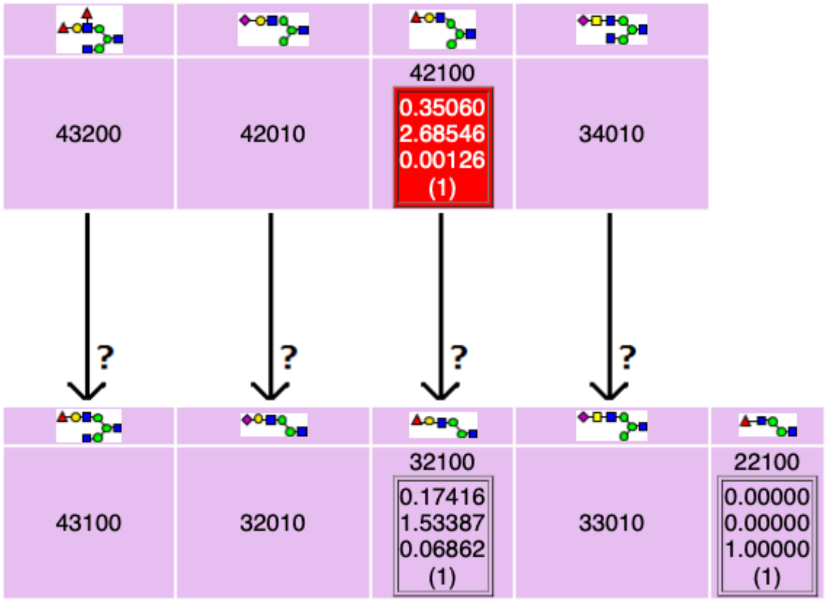
43200
42010
42100
0.35060
2.68546
0.00126
(1)
34010
?
?
?
?
43100
32010
32100
0.17416
1.53387
0.06862
(1)
33010
22100
0.00000
0.00000
1.00000
(1)
