# Supplementary material for: Toolbox Accelerating Glycomics (TAG): Glycan Annotation from MALDI-TOF MS Spectra and Mapping Expression Variation to Biosynthetic Pathways
Source: Biomolecules. 2020 Sep 28;10(10):1383. doi: 10.3390/biom10101383 (PMC7650810; doi:10.3390/biom10101383)

Glycan quantity for each experiment

2: m/z= 1178.4950
(Hex)2 (HexNAc)2

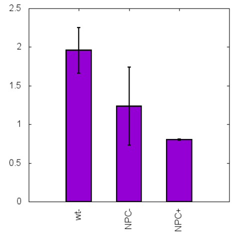

3: m/z= 1340.5478
(Hex)3 (HexNAc)2

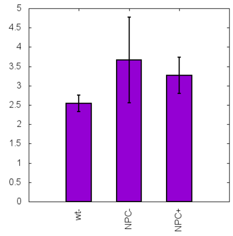

4: m/z= 1502.6006
(Hex)4 (HexNAc)2

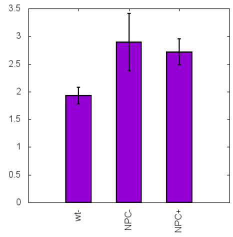

5: m/z= 1664.6534
(Hex)2 + (Man)3(GlcNAc)2

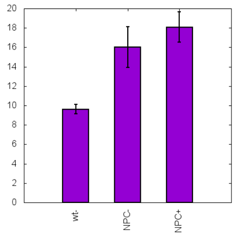

6: m/z= 1162.5001
(Hex)1 (HexNAc)2 (Fuc)1

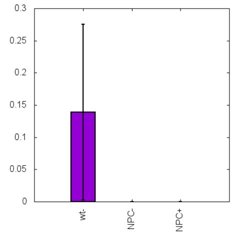

7: m/z= 1324.5529
(Hex)2 (HexNAc)2 (Fuc)1

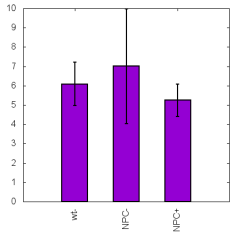

8: m/z= 1486.6057
(Hex)3 (HexNAc)2 (Fuc)1

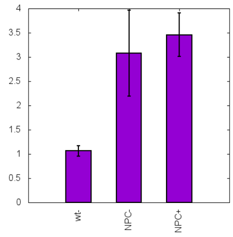

9: m/z= 1648.6585
(Hex)4 (HexNAc)2 (Fuc)1

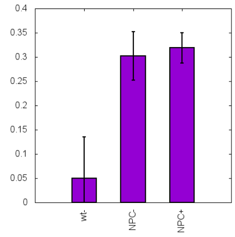

10: m/z= 1810.7113
(Hex)2 (Fuc)1 + (Man)3(GlcNAc)2

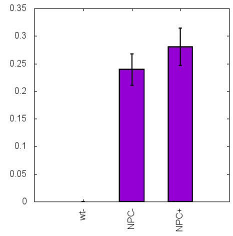

11: m/z= 1826.7062
(Hex)3 + (Man)3(GlcNAc)2

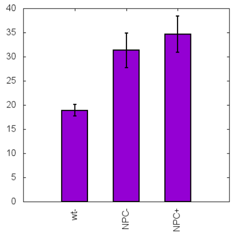

12: m/z= 1988.7590
(Hex)4 + (Man)3(GlcNAc)2

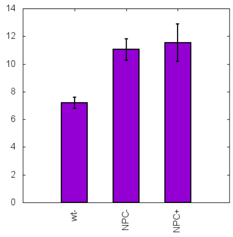

13: m/z= 2150.8118
(Hex)5 + (Man)3(GlcNAc)2

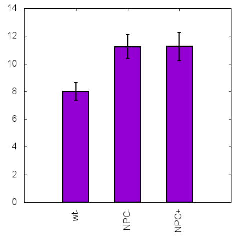

14: m/z= 2312.8646
(Hex)6 + (Man)3(GlcNAc)2

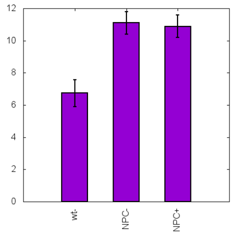

18: m/z= 1543.6271
(HexNAc)1 + (Man)3(GlcNAc)2

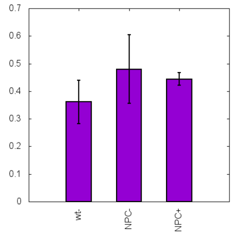

19: m/z= 1746.7064
(HexNAc)2 + (Man)3(GlcNAc)2

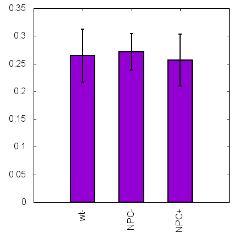

22: m/z= 1705.6799
(Hex)1 (HexNAc)1 + (Man)3(GlcNAc)2

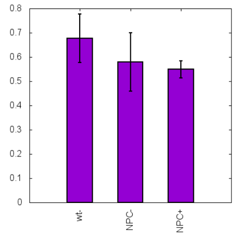

23: m/z= 1908.7592
(Hex)1 (HexNAc)2 + (Man)3(GlcNAc)2

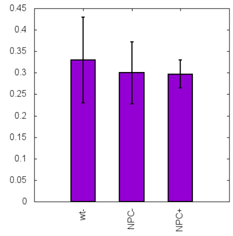

26: m/z= 1867.7327
(Hex)2 (HexNAc)1 + (Man)3(GlcNAc)2

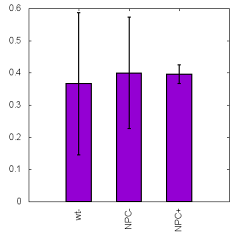

27: m/z= 2070.8120
(Hex)2 (HexNAc)2 + (Man)3(GlcNAc)2

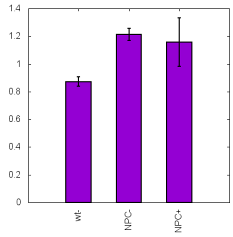

30: m/z= 2029.7855
(Hex)3 (HexNAc)1 + (Man)3(GlcNAc)2

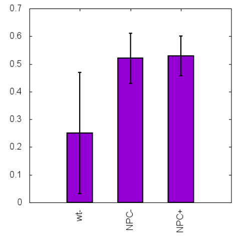

32: m/z= 2435.9441
(Hex)3 (HexNAc)3 + (Man)3(GlcNAc)2

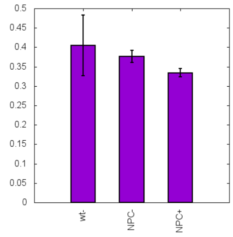

38: m/z= 1689.6850
(HexNAc)1 (Fuc)1 + (Man)3(GlcNAc)2

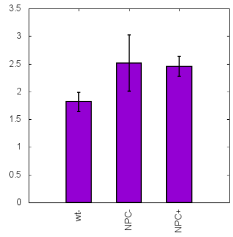

39: m/z= 1892.7643
(HexNAc)2 (Fuc)1 + (Man)3(GlcNAc)2

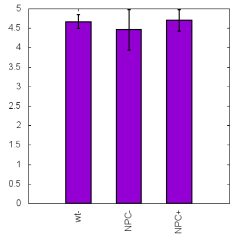

40: m/z= 2095.8436
(HexNAc)3 (Fuc)1 + (Man)3(GlcNAc)2

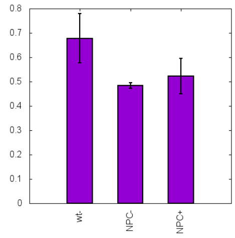

41: m/z= 2298.9229
(HexNAc)4 (Fuc)1 + (Man)3(GlcNAc)2

42: m/z= 1851.7378
(Hex)1 (HexNAc)1 (Fuc)1 + (Man)3(GlcNAc)2

43: m/z= 2054.8171
(Hex)1 (HexNAc)2 (Fuc)1 + (Man)3(GlcNAc)2

44: m/z= 2257.8964
(Hex)1 (HexNAc)3 (Fuc)1 + (Man)3(GlcNAc)2

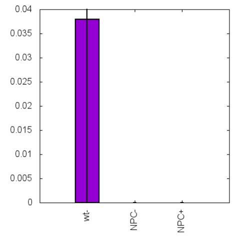

46: m/z= 2013.7906
(Hex)2 (HexNAc)1 (Fuc)1 + (Man)3(GlcNAc)2

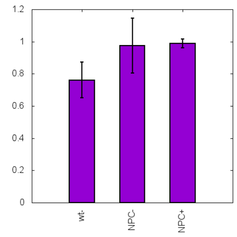

47: m/z= 2216.8699
(Hex)2 (HexNAc)2 (Fuc)1 + (Man)3(GlcNAc)2

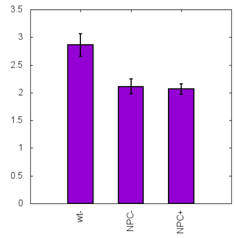

48: m/z= 2419.9492
(Hex)2 (HexNAc)3 (Fuc)1 + (Man)3(GlcNAc)2

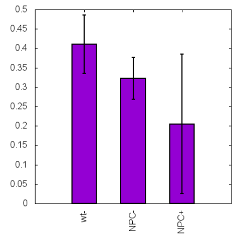

50: m/z= 2175.8434
(Hex)3 (HexNAc)1 (Fuc)1 + (Man)3(GlcNAc)2

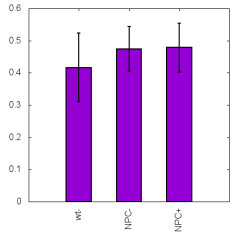

52: m/z= 2582.0020
(Hex)3 (HexNAc)3 (Fuc)1 + (Man)3(GlcNAc)2

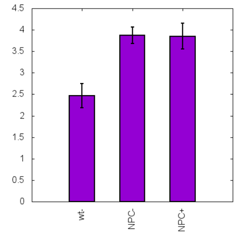

57: m/z= 2947.1341
(Hex)4 (HexNAc)4 (Fuc)1 + (Man)3(GlcNAc)2

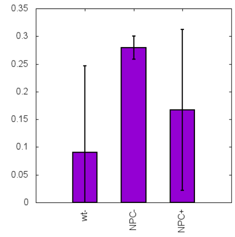

88: m/z= 2010.7909
(Hex)1 (HexNAc)1 (NeuAc)1 + (Man)3(GlcNAc)2

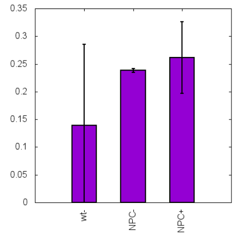

92: m/z= 2172.8437
(Hex)2 (HexNAc)1 (NeuAc)1 + (Man)3(GlcNAc)2

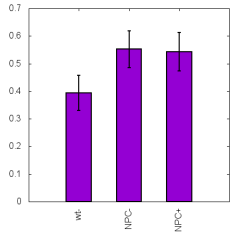

93: m/z= 2375.9230
(Hex)2 (HexNAc)2 (NeuAc)1 + (Man)3(GlcNAc)2

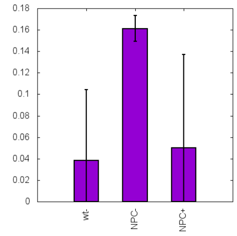

94: m/z= 2579.0023
(Hex)2 (HexNAc)3 (NeuAc)1 + (Man)3(GlcNAc)2

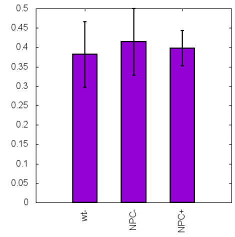

96: m/z= 2334.8965
(Hex)3 (HexNAc)1 (NeuAc)1 + (Man)3(GlcNAc)2

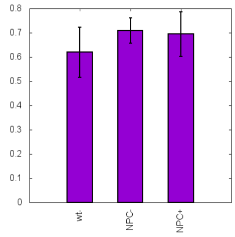

97: m/z= 2537.9758
(Hex)3 (HexNAc)2 (NeuAc)1 + (Man)3(GlcNAc)2

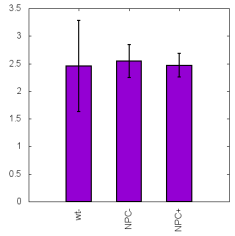

98: m/z= 2741.0551
(Hex)3 (HexNAc)3 (NeuAc)1 + (Man)3(GlcNAc)2

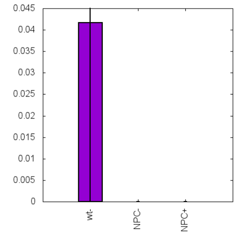

104: m/z= 2156.8488
(Hex)1 (HexNAc)1 (Fuc)1 (NeuAc)1 + (Man)3(GlcNAc)2

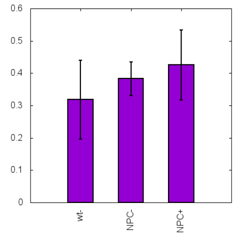

105: m/z= 2359.9281
(Hex)1 (HexNAc)2 (Fuc)1 (NeuAc)1 + (Man)3(GlcNAc)2

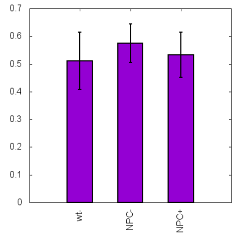

108: m/z= 2318.9016
(Hex)2 (HexNAc)1 (Fuc)1 (NeuAc)1 + (Man)3(GlcNAc)2

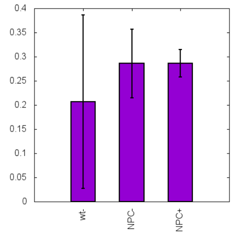

109: m/z= 2521.9809
(Hex)2 (HexNAc)2 (Fuc)1 (NeuAc)1 + (Man)3(GlcNAc)2

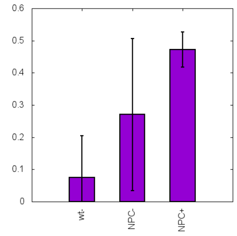

114: m/z= 2887.1130
(Hex)3 (HexNAc)3 (Fuc)1 (NeuAc)1 + (Man)3(GlcNAc)2

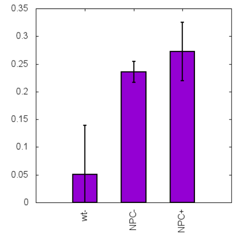

119: m/z= 3252.2451
(Hex)4 (HexNAc)4 (Fuc)1 (NeuAc)1 + (Man)3(GlcNAc)2

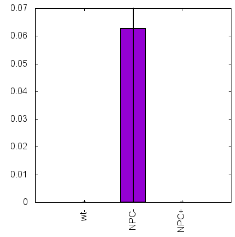

151: m/z= 2417.9335
(Hex)2 (HexNAc)2 (NeuAc)1 (Ac)1 + (Man)3(GlcNAc)2

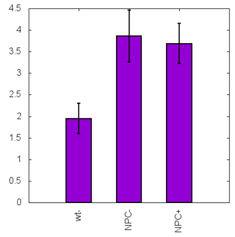

204: m/z= 2681.0340
(Hex)2 (HexNAc)2 (NeuAc)2 + (Man)3(GlcNAc)2

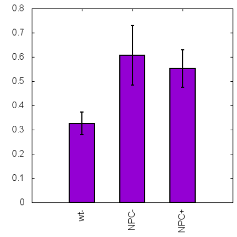

207: m/z= 2843.0868
(Hex)3 (HexNAc)2 (NeuAc)2 + (Man)3(GlcNAc)2

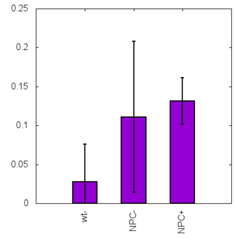

208: m/z= 3046.1661
(Hex)3 (HexNAc)3 (NeuAc)2 + (Man)3(GlcNAc)2

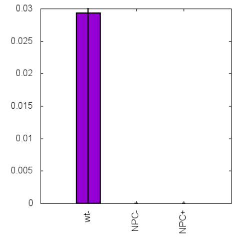

211: m/z= 3208.2189
(Hex)4 (HexNAc)3 (NeuAc)2 + (Man)3(GlcNAc)2

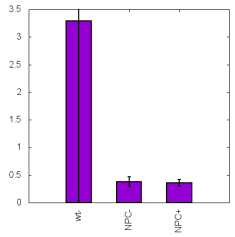

213: m/z= 2827.0919
(Hex)2 (HexNAc)2 (Fuc)1 (NeuAc)2 + (Man)3(GlcNAc)2

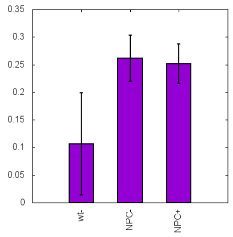

217: m/z= 3192.2240
(Hex)3 (HexNAc)3 (Fuc)1 (NeuAc)2 + (Man)3(GlcNAc)2

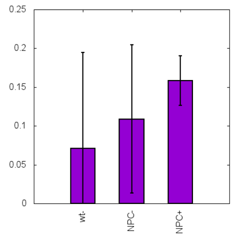

221: m/z= 3557.3561
(Hex)4 (HexNAc)4 (Fuc)1 (NeuAc)2 + (Man)3(GlcNAc)2

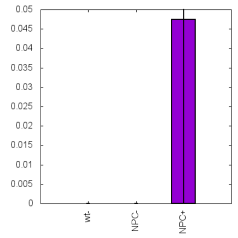

306: m/z= 3351.2771
(Hex)3 (HexNAc)3 (NeuAc)3 + (Man)3(GlcNAc)2

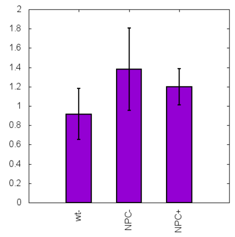

310: m/z= 3497.3350
(Hex)3 (HexNAc)3 (Fuc)1 (NeuAc)3 + (Man)3(GlcNAc)2

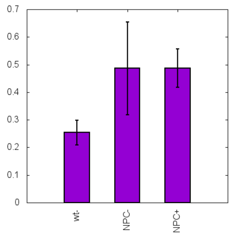

313: m/z= 3862.4671
(Hex)4 (HexNAc)4 (Fuc)1 (NeuAc)3 + (Man)3(GlcNAc)2

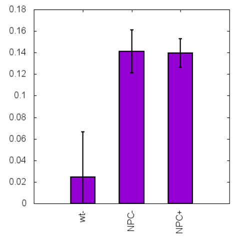

371: m/z= 2391.9179
(Hex)2 (HexNAc)2 (NeuGc)1 + (Man)3(GlcNAc)2

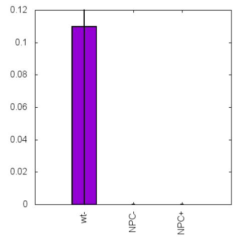

382: m/z= 2172.8437
(Hex)1 (HexNAc)1 (Fuc)1 (NeuGc)1 + (Man)3(GlcNAc)2

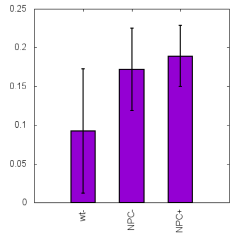

383: m/z= 2375.9230
(Hex)1 (HexNAc)2 (Fuc)1 (NeuGc)1 + (Man)3(GlcNAc)2

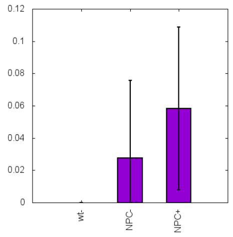

384: m/z= 2579.0023
(Hex)1 (HexNAc)3 (Fuc)1 (NeuGc)1 + (Man)3(GlcNAc)2

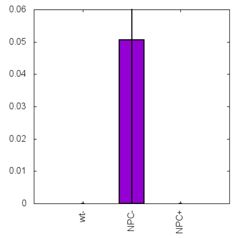

386: m/z= 2334.8965
(Hex)2 (HexNAc)1 (Fuc)1 (NeuGc)1 + (Man)3(GlcNAc)2

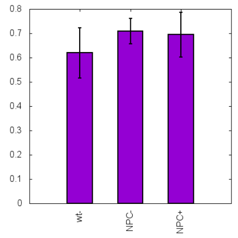

387: m/z= 2537.9758
(Hex)2 (HexNAc)2 (Fuc)1 (NeuGc)1 + (Man)3(GlcNAc)2

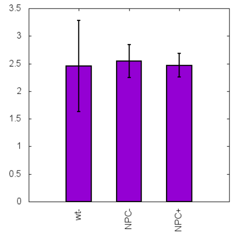

388: m/z= 2741.0551
(Hex)2 (HexNAc)3 (Fuc)1 (NeuGc)1 + (Man)3(GlcNAc)2

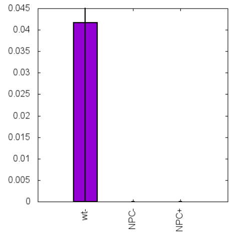

401: m/z= 2887.1130
(Hex)2 (HexNAc)3 (Fuc)2 (NeuGc)1 + (Man)3(GlcNAc)2

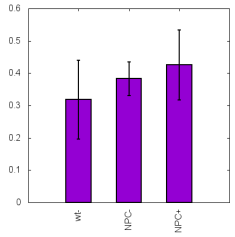

406: m/z= 3252.2451
(Hex)3 (HexNAc)4 (Fuc)2 (NeuGc)1 + (Man)3(GlcNAc)2

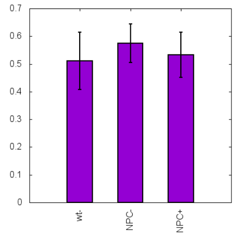

428: m/z= 2230.8491
(Hex)2 (HexNAc)1 (NeuGc)1 (Ac)1 + (Man)3(GlcNAc)2

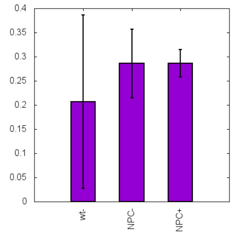

441: m/z= 2417.9335
(Hex)1 (HexNAc)2 (Fuc)1 (NeuGc)1 (Ac)1 + (Man)3(GlcNAc)2

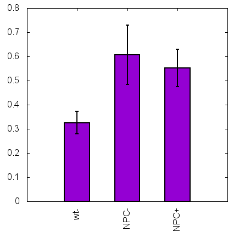

491: m/z= 2843.0868
(Hex)2 (HexNAc)2 (Fuc)1 (NeuAc)1 (NeuGc)1 + (Man)3(GlcNAc)2

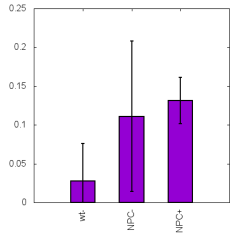

492: m/z= 3046.1661
(Hex)2 (HexNAc)3 (Fuc)1 (NeuAc)1 (NeuGc)1 + (Man)3(GlcNAc)2

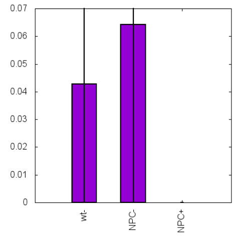

495: m/z= 3208.2189
(Hex)3 (HexNAc)3 (Fuc)1 (NeuAc)1 (NeuGc)1 + (Man)3(GlcNAc)2

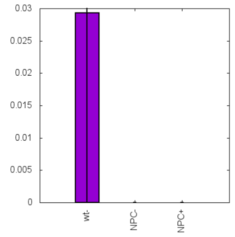

500: m/z= 3192.2240
(Hex)2 (HexNAc)3 (Fuc)2 (NeuAc)1 (NeuGc)1 +

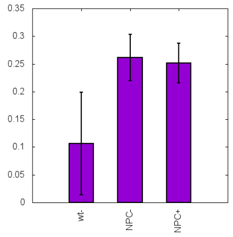

504: m/z= 3557.3561
(Hex)3 (HexNAc)4 (Fuc)2 (NeuAc)1 (NeuGc)1 +

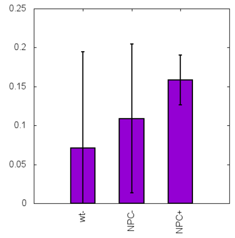

593: m/z= 3862.4671
(Hex)3 (HexNAc)4 (Fuc)2 (NeuAc)2 (NeuGc)1 +

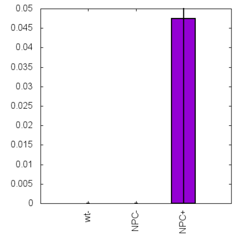

662: m/z= 3208.2189
(Hex)2 (HexNAc)3 (Fuc)2 (NeuGc)2 +

(Man)3(GlcNAc)2

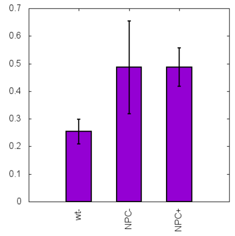

(Man)3(GlcNAc)2

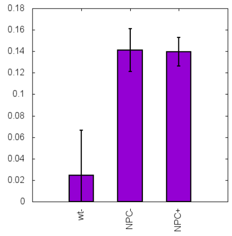

(Man)3(GlcNAc)2

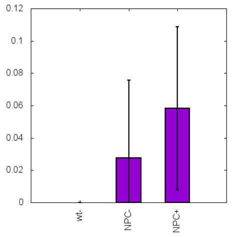

(Man)3(GlcNAc)2

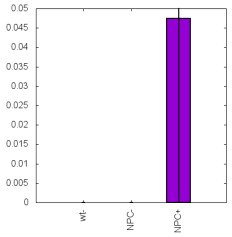

671: m/z= 3557.3561
(Hex)2 (HexNAc)4 (Fuc)3 (NeuGc)2 +
(Man)3(GlcNAc)2

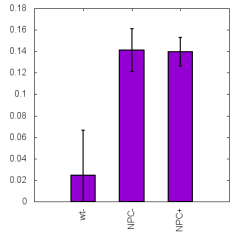

Supplement: Supplementary file 1 [file biomolecules-10-01383-s001.zip › Figure_S1.pdf]
