# Supplementary material for: Toolbox Accelerating Glycomics (TAG): Glycan Annotation from MALDI-TOF MS Spectra and Mapping Expression Variation to Biosynthetic Pathways
Source: Biomolecules. 2020 Sep 28;10(10):1383. doi: 10.3390/biom10101383 (PMC7650810; doi:10.3390/biom10101383)

Glycan quantity for each experiment

2: m/z= 813.3629
(Hex)1 (HexNAc)1

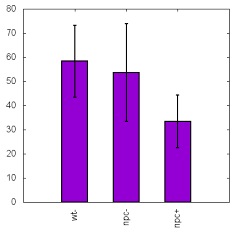

3: m/z= 975.4157
(Hex)2 (HexNAc)1

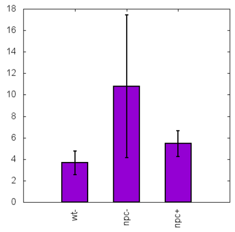

4: m/z= 1137.4685
(Hex)3 (HexNAc)1

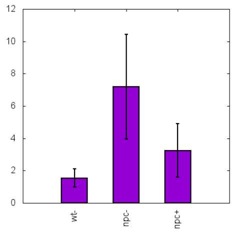

5: m/z= 1299.5213
(Hex)4 (HexNAc)1

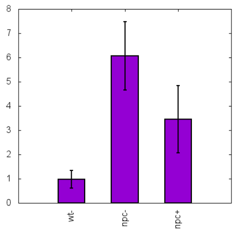

6: m/z= 1461.5741
(Hex)2 + (Man)3(GlcNAc)1

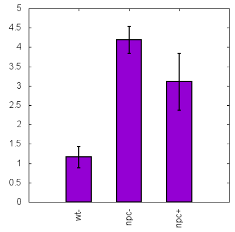

8: m/z= 1016.4422
(Hex)1 (HexNAc)2

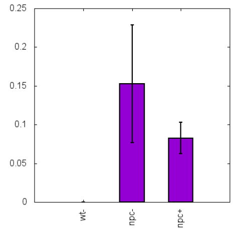

9: m/z= 1178.4950
(Hex)2 (HexNAc)2

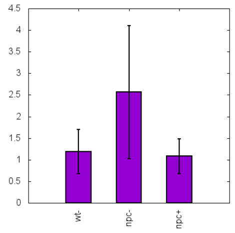

10: m/z= 1340.5478
(Hex)3 (HexNAc)2

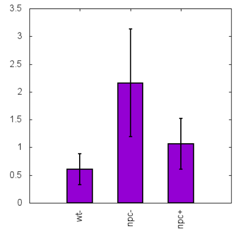

11: m/z= 1502.6006
(Hex)4 (HexNAc)2

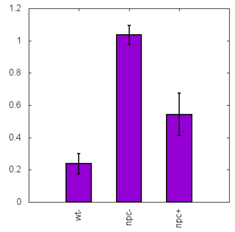

12: m/z= 1664.6534
(Hex)2 + (Man)3(GlcNAc)2

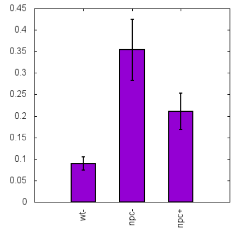

20: m/z= 1486.6057
(Hex)3 (HexNAc)2 (Fuc)1

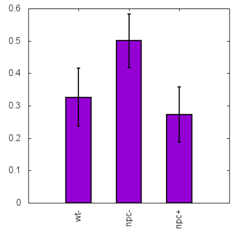

21: m/z= 1648.6585
(Hex)4 (HexNAc)2 (Fuc)1

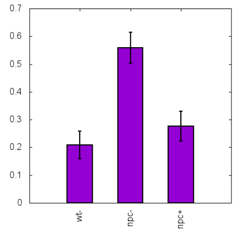

23: m/z= 1623.6269
(Hex)3 + (Man)3(GlcNAc)1

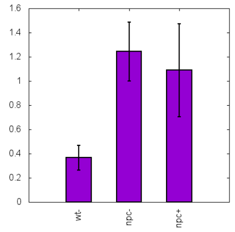

24: m/z= 1785.6797
(Hex)4 + (Man)3(GlcNAc)1

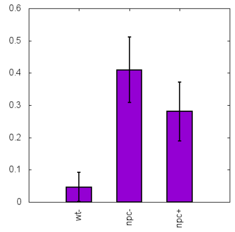

25: m/z= 1947.7325
(Hex)5 + (Man)3(GlcNAc)1

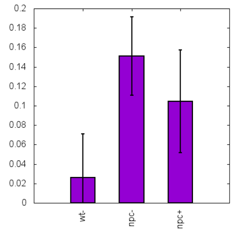

26: m/z= 2109.7853
(Hex)6 + (Man)3(GlcNAc)1

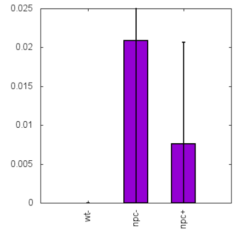

27: m/z= 1826.7062
(Hex)3 + (Man)3(GlcNAc)2

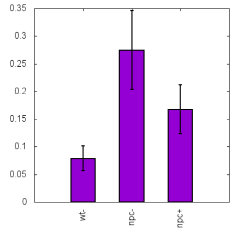

28: m/z= 1988.7590
(Hex)4 + (Man)3(GlcNAc)2

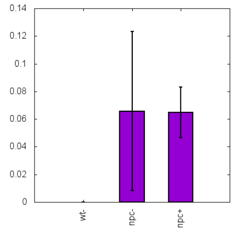

29: m/z= 2150.8118
(Hex)5 + (Man)3(GlcNAc)2

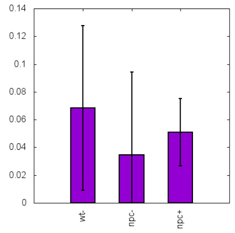

37: m/z= 1340.5478
(HexNAc)1 + (Man)3(GlcNAc)1

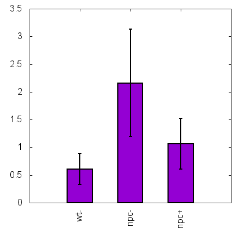

38: m/z= 1543.6271
(HexNAc)1 + (Man)3(GlcNAc)2

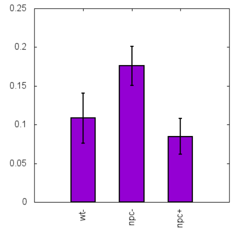

39: m/z= 1543.6271
(HexNAc)2 + (Man)3(GlcNAc)1

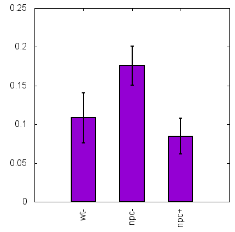

40: m/z= 1746.7064
(HexNAc)2 + (Man)3(GlcNAc)2

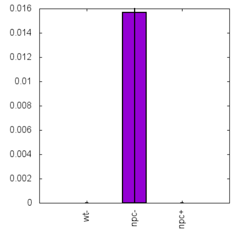

41: m/z= 1746.7064
(HexNAc)3 + (Man)3(GlcNAc)1

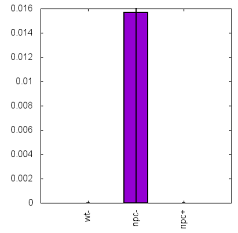

45: m/z= 1502.6006
(Hex)1 (HexNAc)1 + (Man)3(GlcNAc)1

46: m/z= 1705.6799
(Hex)1 (HexNAc)1 + (Man)3(GlcNAc)2

47: m/z= 1705.6799
(Hex)1 (HexNAc)2 + (Man)3(GlcNAc)1

48: m/z= 1908.7592
(Hex)1 (HexNAc)2 + (Man)3(GlcNAc)2

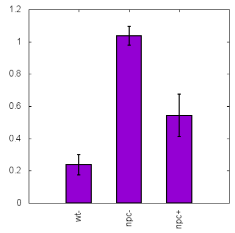

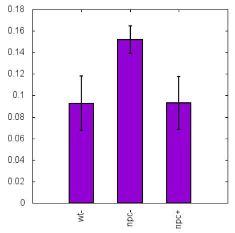

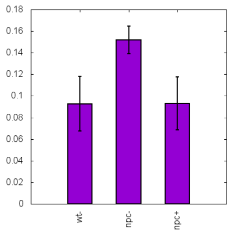

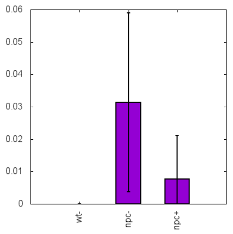

49: m/z= 1908.7592
(Hex)1 (HexNAc)3 + (Man)3(GlcNAc)1

53: m/z= 1664.6534
(Hex)2 (HexNAc)1 + (Man)3(GlcNAc)1

54: m/z= 1867.7327
(Hex)2 (HexNAc)1 + (Man)3(GlcNAc)2

55: m/z= 1867.7327
(Hex)2 (HexNAc)2 + (Man)3(GlcNAc)1

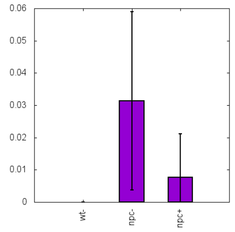

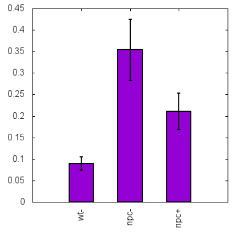

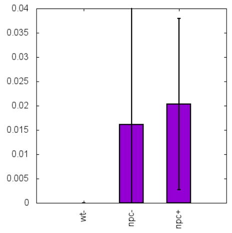

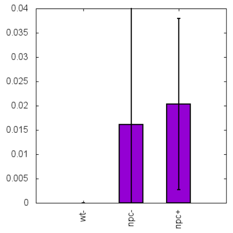

56: m/z= 2070.8120
(Hex)2 (HexNAc)2 + (Man)3(GlcNAc)2

57: m/z= 2070.8120
(Hex)2 (HexNAc)3 + (Man)3(GlcNAc)1

61: m/z= 1826.7062
(Hex)3 (HexNAc)1 + (Man)3(GlcNAc)1

66: m/z= 2435.9441
(Hex)3 (HexNAc)3 + (Man)3(GlcNAc)2

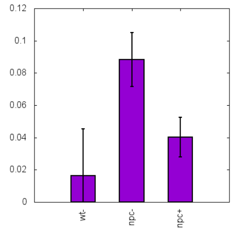

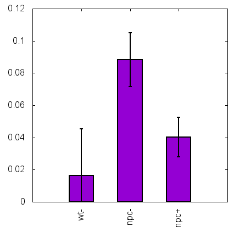

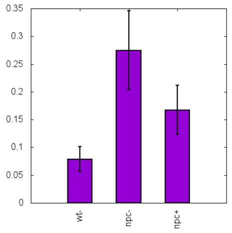

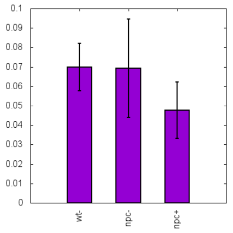

67: m/z= 2435.9441
(Hex)3 (HexNAc)4 + (Man)3(GlcNAc)1

69: m/z= 1988.7590
(Hex)4 (HexNAc)1 + (Man)3(GlcNAc)1

77: m/z= 1486.6057
(HexNAc)1 (Fuc)1 + (Man)3(GlcNAc)1

85: m/z= 1648.6585
(Hex)1 (HexNAc)1 (Fuc)1 + (Man)3(GlcNAc)1

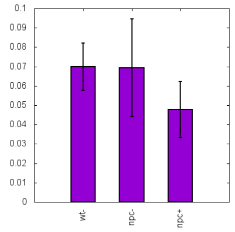

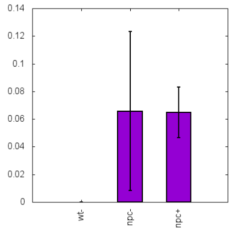

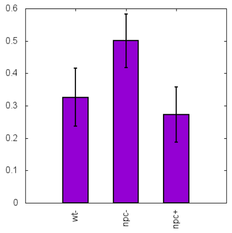

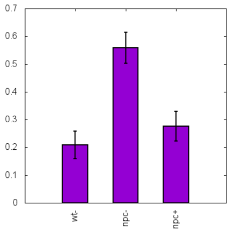

86: m/z= 1851.7378
(Hex)1 (HexNAc)1 (Fuc)1 + (Man)3(GlcNAc)2

87: m/z= 1851.7378
(Hex)1 (HexNAc)2 (Fuc)1 + (Man)3(GlcNAc)1

126: m/z= 2159.8485
(Hex)2 (HexNAc)1 (Fuc)2 + (Man)3(GlcNAc)2

177: m/z= 1807.7116
(Hex)1 (HexNAc)1 (NeuAc)1 + (Man)3(GlcNAc)1

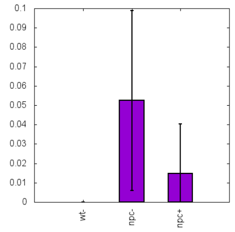

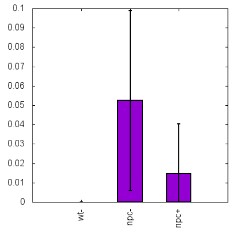

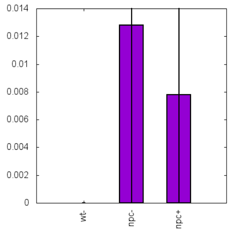

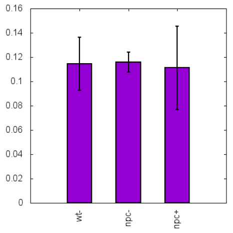

178: m/z= 2010.7909
(Hex)1 (HexNAc)1 (NeuAc)1 + (Man)3(GlcNAc)2

179: m/z= 2010.7909
(Hex)1 (HexNAc)2 (NeuAc)1 + (Man)3(GlcNAc)1

180: m/z= 2213.8702
(Hex)1 (HexNAc)2 (NeuAc)1 + (Man)3(GlcNAc)2

181: m/z= 2213.8702
(Hex)1 (HexNAc)3 (NeuAc)1 + (Man)3(GlcNAc)1

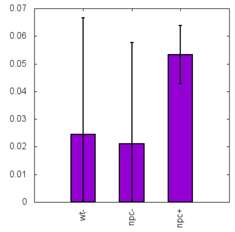

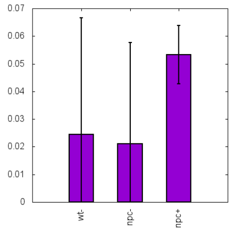

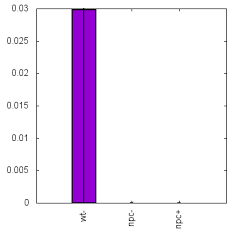

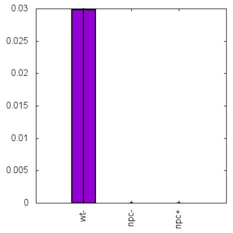

186: m/z= 2172.8437
(Hex)2 (HexNAc)1 (NeuAc)1 + (Man)3(GlcNAc)2

187: m/z= 2172.8437
(Hex)2 (HexNAc)2 (NeuAc)1 + (Man)3(GlcNAc)1

409: m/z= 2477.9547
(Hex)2 (HexNAc)2 (NeuAc)2 + (Man)3(GlcNAc)1

477: m/z= 2519.9652
(Hex)2 (HexNAc)2 (NeuAc)2 (Ac)1 +

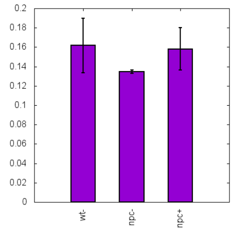

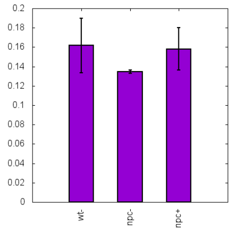

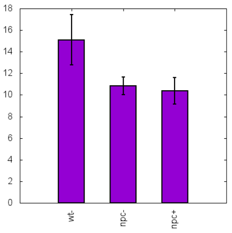

(Man)3(GlcNAc)1

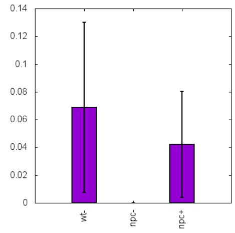

766: m/z= 2172.8437
(Hex)1 (HexNAc)1 (Fuc)1 (NeuGc)1 + (Man)3(GlcNAc)2

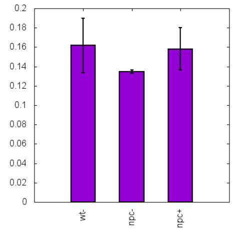

767: m/z= 2172.8437
(Hex)1 (HexNAc)2 (Fuc)1 (NeuGc)1 + (Man)3(GlcNAc)1

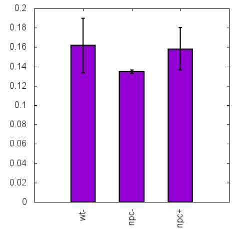

1757: m/z= 1178.5130
(HexNAc)1 + (Man)2(GlcNAc)1

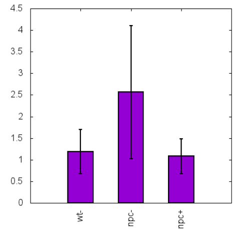

1759: m/z= 1137.4865
(Hex)1 + (Man)2(GlcNAc)1

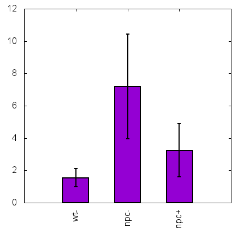

1760: m/z= 1340.5838
(Hex)1 + (Man)2(GlcNAc)2

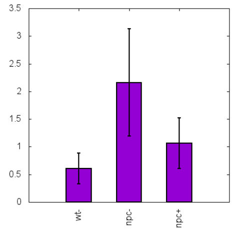

1761: m/z= 1340.5658
(Hex)1 (HexNAc)1 + (Man)2(GlcNAc)1

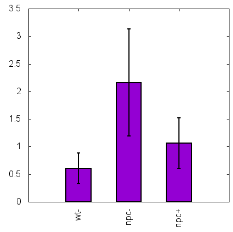

1762: m/z= 1543.6631
(Hex)1 (HexNAc)1 + (Man)2(GlcNAc)2

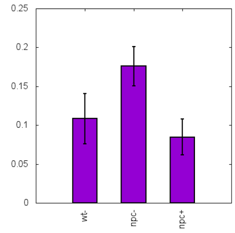

1767: m/z= 1645.6768
(Hex)1 (HexNAc)1 (NeuAc)1 + (Man)2(GlcNAc)1

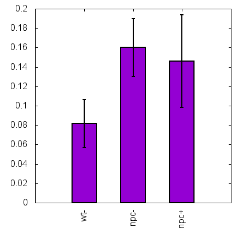

Supplement: Supplementary file 1 [file biomolecules-10-01383-s001.zip › Figure_S2.pdf]
