# Supplementary material for: Toolbox Accelerating Glycomics (TAG): Glycan Annotation from MALDI-TOF MS Spectra and Mapping Expression Variation to Biosynthetic Pathways
Source: Biomolecules. 2020 Sep 28;10(10):1383. doi: 10.3390/biom10101383 (PMC7650810; doi:10.3390/biom10101383)
Supplement: Supplementary file 1 [file biomolecules-10-01383-s001.zip › TableS1_and_S2.docx]

**Table S1** **Classification of N-glycans.** Glycan types are indicated by dividing the symbols assigned to term for their key properties by underscore symbol in the abbreviation column. In the first group, PM, HM, and Hy represent the pauci mannose, high-mannose, and hybrid type, respectively, while the others represent complex forms. In the complex type, the first integer designated the number of branches of glycans such as biantennary or tetraantennary, etc.; symbol ‘b’ means the possibility of the bisecting structure; ‘L’ indicates the possibility of the LacdiNAc structure. In the second group, symbol ‘a’ or ‘n’ means whether sialylated or non-sialylated glycan. In last group, symbol ‘F’ and ‘G’ indicate that the glycan is fucosylated and glucosylated HM, respectively.

|  | Type of glycan | Abbreviation | Definition |
| --- | --- | --- | --- |
| 1 | Pauci-mannose | PM | H = 1−4, HN = 2, F = 0, Sia ^1^ = 0 |
| 2 | Pauci-mannose with fucose | PM_F | H = 1−5, HN = 2, F = 1, Sia = 0 |
| 3 | High-mannose | HM | H = 5−9, HN = 2, F = 0, Sia = 0 |
| 4 | High-mannose with glycose | HM_G | H = 10−12, HN = 2, F = 0, Sia = 0 |
| 5 | Mono-antennary neutral glycan | 1_n | H = 0−1, HN = 1, F = 0, Sia = 0 ^2^ |
| 6 | Mono-antennary neutral glycan with fucose | 1_n_F | H = 0−1, HN = 1, F = 1, Sia = 0 ^2^ |
| 7 | Neutral hybrid glycan | Hy_n | H >HN, HN >0, F = 0, Sia = 0 ^2^ |
| 8 | Neutral hybrid glycan with fucose | Hy_n_F | H >HN, HN >0, F >0, Sia = 0 ^2^ |
| 9 | Biantennary neutral glycan | 2_n | H = 0−2, HN = 2, F = 0, Sia = 0 ^2^ |
| 10 | Biantennary neutral glycan with fucose | 2_n_F | H = 0−2, HN = 2, F >0 Sia = 0 ^2^ |
| 11 | Triantennary, bisected biantennary, or LacdiNAc neutral glycan | 3bL_n | H = 0−1, HN = 3, F = 0, Sia = 0 ^2^ |
| 12 | Triantennary, bisected biantennary, or LacdiNAc neutral glycan with fucose | 3bL_n_F | H = 0−1, HN = 3, F >0, Sia = 0 ^2^ |
| 13 | Triantennary or bisected biantennary neutral glycan | 3b_n | H = 2, HN = 3, F = 0, Sia = 0 ^2^ |
| 14 | Triantennary or bisected biantennary neutral glycan with fucose | 3b_n_F | H = 2, HN = 3, F >0, Sia = 0 ^2^ |
| 15 | Triantennary neutral glycan | 3_n | H = 3, HN = 3, F = 0, Sia = 0 ^2^ |
| 16 | Trianntenally neutral glycan with fucose | 3_n_F | H = 3, HN = 3, F >0, Sia = 0 ^2^ |
| 17 | Tetraantennary, bisected triantennary or LacdiNAc neutral glycan | 4bL_n | H = 0−2, HN = 4, F = 0, Sia = 0 ^2^ |
| 18 | Tetraantennary, bisected triantennary or LacdiNAc neutral glycan with fucose | 4bL_n_F | H = 0−2, HN = 4, F >0, Sia = 0 ^2^ |
| 19 | Tetraantennary or bisected triantennary neutral glycan | 4b_n | H = 3, HN = 4, F = 0, Sia = 0 ^2^ |
| 20 | Tetraantennary or bisected triantennary neutral glycan with fucose | 4b_n_F | H = 3, HN = 4, F >0, Sia = 0 ^2^ |
| 21 | Tetraantennary neutral glycan | 4_n | H = 4, HN = 4, F = 0, Sia = 0 ^2^ |
| 22 | Tetraantennary neutral glycan with fucose | 4_n_F | H = 4, HN = 4, F >0, Sia = 0 ^2^ |
| 23 | Monoantennary acid glycan | 1_a | H = 0−1, HN = 1, F = 0, Sia >0 ^2^ |
| 24 | Monoantennary acid glycan with fucose | 1_a_F | H = 0−1, HN = 1, F = 1, Sia >0 ^2^ |
| 25 | Acid hybrid glycan | Hy_a | H >HN, HN >0, F = 0, Sia >0 ^2^ |
| 26 | Acid hybrid glycan with fucose | Hy_a_F | H >HN, HN >0, F >0, Sia >0 ^2^ |
| 27 | Biantennary acid glycan | 2_a | H = 0−2, HN = 2, F = 0, Sia >0 ^2^ |
| 28 | Biantennary LacdiNAc acid glycan | 2L_a | H = 0, HN = 3−4, F = 0, Sia >0 ^2^ |
| 29 | Biantennary LacdiNAc acid glycan with fucose | 2L_a_F | H = 0, HN = 3−4, F >0, Sia >0 ^2^ |
| 30 | Biantennary acid glycan with fucose | 2_a_F | H = 0−2, HN = 2, F >0, Sia >0 ^2^ |
| 31 | Triantennary, bisected biantennary, or LacdiNAc acid glycan | 3bL_a | H = 0−1, HN = 3, F = 0, Sia >0 ^2^ |
| 32 | Triantennary, bisected biantennary, or LacdiNAc acid glycan with fucose | 3bL_a_F | H = 0−1, HN = 3, F >0, Sia >0 ^2^ |
| 33 | Triantennary or bisected biantennary acid glycan | 3b_a | H = 2, HN = 3, F = 0, Sia >0 ^2^ |
| 34 | Triantennary or bisected biantennary acid glycan with fucose | 3b_a_F | H = 2, HN = 3, F >0, Sia >0 ^2^ |
| 35 | Triantennary acid glycan | 3_a | H = 3, HN = 3, F = 0, Sia >0, Sia ≠ 4 ^2^ |
| 36 | Triantennary acid glycan with fucose | 3_a_F | H = 3, HN = 3, F >0, Sia >0, Sia ≠ 4 ^2^ |
| 37 | Tetra-antennary, bisected triantennary, LacdiNAc acid glycan | 4bL_a | H = 0−2, HN = 4, F = 0, Sia >0 ^2^ |
| 38 | Tetra-antennary, bisected triantennary, LacdiNAc acid glycan with fucose | 4bL_a_F | H = 0−2, HN = 4, F >0, Sia >0 ^2^ |
| 39 | Tetra-antennary or bisected triantennary acid glycan | 4b_a | H = 3, HN = 4, F = 0, Sia >0 ^2^ |
| 40 | Tetra-antennary or bisected triantennary acid glycan with fucose | 4b_a_F | H = 3, HN = 4, F >0, Sia >0 ^2^ |
| 41 | Tetraantennary acid glycan | 4_a | H = 4, HN = 4, F = 0, Sia >0 ^2^ |
| 42 | Triantennary FCS glycan | 3_a_FCS | H = 3, HN = 3, Sia = 4 ^2^ |
| 43 | Tetraantennary acid glycan with fucose | 4_a_F | H = 4, HN = 4, F >0, Sia >0 ^2^ |

^1^H, HN, F, and Sia indicate the number of hexoses, N-acetyl hexoses, fucoses (deoxy hexose), and sialic acids. Sia also refers to the summation of the number of N-acetyl neuraminic acid and N-glycolyl neuraminic acid units.

^2^The M3 core (H = 3, HN = 2) is omitted in the definition of these classes. Therefore, the actual number of residues is calculated using H = 3 and HN = 2.

**Table S2 Classification of free N-glycans (FNGs).** The means of symbols in the abbreviation column are the same in Table S1.

|  | Type of glycan | Abbreviation | Definition |
| --- | --- | --- | --- |
| 1 | Pauci-mannose | PM_N1 | H=1−4, HN=1, F=0, Sia^1^=0 |
| 2 | Pauci-mannose with fucose | PM_F_N1 | H=1−5, HN=1, F=1, Sia=0 |
| 3 | High-mannose | HM_N1 | H=5−9, HN=1, F=0, Sia=0 |
| 4 | High-mannose with glycose | HM_G_N1 | H=10−12, HN=1, F=0, Sia=0 |
| 5 | Pauci-mannose | PM_N1 | H=1−4, HN=2, F=0, Sia^1^=0 |
| 6 | Pauci-mannose with fucose | PM_F_N1 | H=1−5, HN=2, F=1, Sia=0 |
| 7 | High-mannose | HM_N1 | H=5−9, HN=2, F=0, Sia=0 |
| 8 | High-mannose with glycose | HM_G_N1 | H=10−12, HN=2, F=0, Sia=0 |
| 9 | Mono-antennary neutral glycan | 1_n_N1 | H=0−1, HN=1, F=0, Sia=0 ^2^ |
| 10 | Mono-antennary neutral glycan with fucose | 1_n_F_N1 | H=0−1, HN=1, F=1, Sia=0 ^2^ |
| 11 | Neutral hybrid glycan | Hy_n_N1 | H>HN, HN>0, F=0, Sia=0 ^2^ |
| 12 | Neutral hybrid glycan with fucose | Hy_n_F_N1 | H>HN, HN>0, F>0, Sia=0 ^2^ |
| 13 | Biantennary neutral glycan | 2_n_N1 | H=0−2, HN=2, F=0, Sia=0 ^2^ |
| 14 | Biantennary neutral glycan with fucose | 2_n_F_N1 | H=0−2, HN=2, F>0 Sia=0 ^2^ |
| 15 | Triantennary, bisected biantennary, or acdiNAc neutral glycan | 3bL_n_N1 | H=0−1, HN=3, F=0, Sia=0 ^2^ |
| 16 | Triantennary, bisected biantennary, or LacdiNAc neutral glycan with fucose | 3bL_n_F_N1 | H=0−1, HN=3, F>0, Sia=0 ^2^ |
| 17 | Triantennary or bisected biantennary neutral glycan | 3b_n_N1 | H=2, HN=3, F=0, Sia=0 ^2^ |
| 18 | Triantennary or bisected biantennary neutral glycan with fucose | 3b_n_F_N1 | H=2, HN=3, F>0, Sia=0 ^2^ |
| 19 | Triantennary neutral glycan | 3_n_N1 | H=3, HN=3, F=0, Sia=0 ^2^ |
| 20 | Trianntenally neutral glycan with fucose | 3_n_F_N1 | H=3, HN=3, F>0, Sia=0 ^2^ |
| 21 | Tetraantennary, bisected triantennary or LacdiNAc neutral glycan | 4bL_n_N1 | H=0−2, HN=4, F=0, Sia=0 ^2^ |
| 22 | Tetraantennary, bisected triantennary or LacdiNAc neutral glycan with fucose | 4bL_n_F_N1 | H=0−2, HN=4, F>0, Sia=0 ^2^ |
| 23 | Tetraantennary or bisected triantennary neutral glycan | 4b_n_N1 | H=3, HN=4, F=0, Sia=0 ^2^ |
| 24 | Tetraantennary or bisected triantennary neutral glycan with fucose | 4b_n_F_N1 | H=3, HN=4, F>0, Sia=0 ^2^ |
| 25 | Tetraantennary neutral glycan | 4_n_N1 | H=4, HN=4, F=0, Sia=0 ^2^ |
| 26 | Tetraantennary neutral glycan with fucose | 4_n_F_N1 | H=4, HN=4, F>0, Sia=0 ^2^ |
| 27 | Monoantennary acid glycan | 1_a_N1 | H=0−1, HN=1, F=0, Sia>0 ^2^ |
| 28 | Monoantennary acid glycan with fucose | 1_a_F_N1 | H=0−1, HN=1, F=1, Sia>0 ^2^ |
| 29 | Acid hybrid glycan | Hy_a_N1 | H>HN, HN>0, F=0, Sia>0 ^2^ |
| 30 | Acid hybrid glycan with fucose | Hy_a_F_N1 | H>HN, HN>0, F>0, Sia>0 ^2^ |
| 31 | Biantennary acid glycan | 2_a_N1 | H=0−2, HN=2, F=0, Sia>0 ^2^ |
| 32 | Biantennary acid glycan with fucose | 2_a_F_N1 | H=0−2, HN=2, F>0, Sia>0 ^2^ |
| 33 | Triantennary, bisected biantennary, or LacdiNAc acid glycan | 3bL_a_N1 | H=0−1, HN=3, F=0, Sia>0 ^2^ |
| 34 | Triantennary, bisected biantennary, or LacdiNAc acid glycan with fucose | 3bL_a_F_N1 | H=0−1, HN=3, F>0, Sia>0 ^2^ |
| 35 | Triantennary or bisected biantennary acid glycan | 3b_a_N1 | H=2, HN=3, F=0, Sia>0 ^2^ |
| 36 | Triantennary or bisected biantennary acid glycan with fucose | 3b_a_F_N1 | H=2, HN=3, F>0, Sia>0 ^2^ |
| 37 | Triantennary acid glycan | 3_a_N1 | H=3, HN=3, F=0, Sia>0, Sia≠4 ^2^ |
| 38 | Triantennary acid glycan with fucose | 3_a_F_N1 | H=3, HN=3, F>0, Sia>0, Sia≠4 ^2^ |
| 39 | Tetra-antennary, bisected triantennary, LacdiNAc acid glycan | 4bL_a_N1 | H=0−2, HN=4, F=0, Sia>0 ^2^ |
| 40 | Tetra-antennary, bisected triantennary, LacdiNAc acid glycan with fucose | 4bL_a_F_N1 | H=0−2, HN=4, F>0, Sia>0 ^2^ |
| 41 | Tetra-antennary or bisected triantennary acid glycan | 4b_a_N1 | H=3, HN=4, F=0, Sia>0 ^2^ |
| 42 | Tetra-antennary or bisected triantennary acid glycan with fucose | 4b_a_F_N1 | H=3, HN=4, F>0, Sia>0 ^2^ |
| 43 | Tetraantennary acid glycan | 4_a_N1 | H=4, HN=4, F=0, Sia>0 ^2^ |
| 44 | Triantennary FCS glycan | 3_a_FCS_N1 | H=3, HN=3, Sia=4 ^2^ |
| 45 | Tetraantennary acid glycan with fucose | 4_a_F_N1 | H=4, HN=4, F>0, Sia>0 ^2^ |
| 46 | Mono-antennary neutral glycan | 1_n_N2 | H=0−1, HN=1, F=0, Sia=0 ^3^ |
| 47 | Mono-antennary neutral glycan with fucose | 1_n_F_N2 | H=0−1, HN=1, F=1, Sia=0 ^3^ |
| 48 | Neutral hybrid glycan | Hy_n_N2 | H>HN, HN>0, F=0, Sia=0 ^3^ |
| 49 | Neutral hybrid glycan with fucose | Hy_n_F_N2 | H>HN, HN>0, F>0, Sia=0 ^3^ |
| 50 | Biantennary neutral glycan | 2_n_N2 | H=0−2, HN=2, F=0, Sia=0 ^3^ |
| 50 | Biantennary neutral glycan with fucose | 2_n_F_N2 | H=0−2, HN=2, F>0 Sia=0 ^3^ |
| 52 | Triantennary, bisected biantennary, or acdiNAc neutral glycan | 3bL_n_N2 | H=0−1, HN=3, F=0, Sia=0 ^3^ |
| 53 | Triantennary, bisected biantennary, or LacdiNAc neutral glycan with fucose | 3bL_n_F_N2 | H=0−1, HN=3, F>0, Sia=0 ^3^ |
| 54 | Triantennary or bisected biantennary neutral glycan | 3b_n_N2 | H=2, HN=3, F=0, Sia=0 ^3^ |
| 55 | Triantennary or bisected biantennary neutral glycan with fucose | 3b_n_F_N2 | H=2, HN=3, F>0, Sia=0 ^3^ |
| 56 | Triantennary neutral glycan | 3_n_N2 | H=3, HN=3, F=0, Sia=0 ^3^ |
| 57 | Trianntenally neutral glycan with fucose | 3_n_F_N2 | H=3, HN=3, F>0, Sia=0 ^3^ |
| 58 | Tetraantennary, bisected triantennary or LacdiNAc neutral glycan | 4bL_n_N2 | H=0−2, HN=4, F=0, Sia=0 ^3^ |
| 59 | Tetraantennary, bisected triantennary or LacdiNAc neutral glycan with fucose | 4bL_n_F_N2 | H=0−2, HN=4, F>0, Sia=0 ^3^ |
| 60 | Tetraantennary or bisected triantennary neutral glycan | 4b_n_N2 | H=3, HN=4, F=0, Sia=0 ^3^ |
| 61 | Tetraantennary or bisected triantennary neutral glycan with fucose | 4b_n_F_N2 | H=3, HN=4, F>0, Sia=0 ^3^ |
| 62 | Tetraantennary neutral glycan | 4_n_N2 | H=4, HN=4, F=0, Sia=0 ^3^ |
| 63 | Tetraantennary neutral glycan with fucose | 4_n_F_N2 | H=4, HN=4, F>0, Sia=0 ^3^ |
| 64 | Monoantennary acid glycan | 1_a_N2 | H=0−1, HN=1, F=0, Sia>0 ^3^ |
| 65 | Monoantennary acid glycan with fucose | 1_a_F_N2 | H=0−1, HN=1, F=1, Sia>0 ^3^ |
| 66 | Acid hybrid glycan | Hy_a_N2 | H>HN, HN>0, F=0, Sia>0 ^3^ |
| 67 | Acid hybrid glycan with fucose | Hy_a_F_N2 | H>HN, HN>0, F>0, Sia>0 ^3^ |
| 68 | Biantennary acid glycan | 2_a_N2 | H=0−2, HN=2, F=0, Sia>0 ^3^ |
| 69 | Biantennary acid glycan with fucose | 2_a_F_N2 | H=0−2, HN=2, F>0, Sia>0 ^3^ |
| 70 | Triantennary, bisected biantennary, or LacdiNAc acid glycan | 3bL_a_N2 | H=0−1, HN=3, F=0, Sia>0 ^3^ |
| 71 | Triantennary, bisected biantennary, or LacdiNAc acid glycan with fucose | 3bL_a_F_N2 | H=0−1, HN=3, F>0, Sia>0 ^3^ |
| 72 | Triantennary or bisected biantennary acid glycan | 3b_a_N2 | H=2, HN=3, F=0, Sia>0 ^3^ |
| 73 | Triantennary or bisected biantennary acid glycan with fucose | 3b_a_F_N2 | H=2, HN=3, F>0, Sia>0 ^3^ |
| 74 | Triantennary acid glycan | 3_a_N2 | H=3, HN=3, F=0, Sia>0, Sia≠4 ^3^ |
| 75 | Triantennary acid glycan with fucose | 3_a_F_N2 | H=3, HN=3, F>0, Sia>0, Sia≠4 ^3^ |
| 76 | Tetra-antennary, bisected triantennary, LacdiNAc acid glycan | 4bL_a_N2 | H=0−2, HN=4, F=0, Sia>0 ^3^ |
| 77 | Tetra-antennary, bisected triantennary, LacdiNAc acid glycan with fucose | 4bL_a_F_N2 | H=0−2, HN=4, F>0, Sia>0 ^3^ |
| 78 | Tetra-antennary or bisected triantennary acid glycan | 4b_a_N2 | H=3, HN=4, F=0, Sia>0 ^3^ |
| 79 | Tetra-antennary or bisected triantennary acid glycan with fucose | 4b_a_F_N2 | H=3, HN=4, F>0, Sia>0 ^3^ |
| 80 | Tetraantennary acid glycan | 4_a_N2 | H=4, HN=4, F=0, Sia>0 ^3^ |
| 81 | Triantennary FCS glycan | 3_a_FCS_N2 | H=3, HN=3, Sia=4 ^3^ |
| 82 | Tetraantennary acid glycan with fucose | 4_a_F_N2 | H=4, HN=4, F>0, Sia>0 ^3^ |

^1^H, HN, F and Sia indicates the number of hexoses, N-acetyl hexoses, fucoses (deoxy hexose), and sialic acids. The Sia also refers to the summation of the number of N-acetyl neuraminic acid and N-glycolyl neuraminic acid units.

^2^The N1 core (H=3, HN=1) is omitted in the definition of these classes. Therefore, the actual number of residues is calculated using H=3 and HN=1.

^3^The N2 core (H=3, HN=2) is omitted in the definition of these classes. Therefore, the actual number of residues is calculated using H=3 and HN=2
