# Supplementary figures and images for: Toolbox Accelerating Glycomics (TAG): Glycan Annotation from MALDI-TOF MS Spectra and Mapping Expression Variation to Biosynthetic Pathways
Source: Biomolecules. 2020 Sep 28;10(10):1383. doi: 10.3390/biom10101383 (PMC7650810; doi:10.3390/biom10101383)

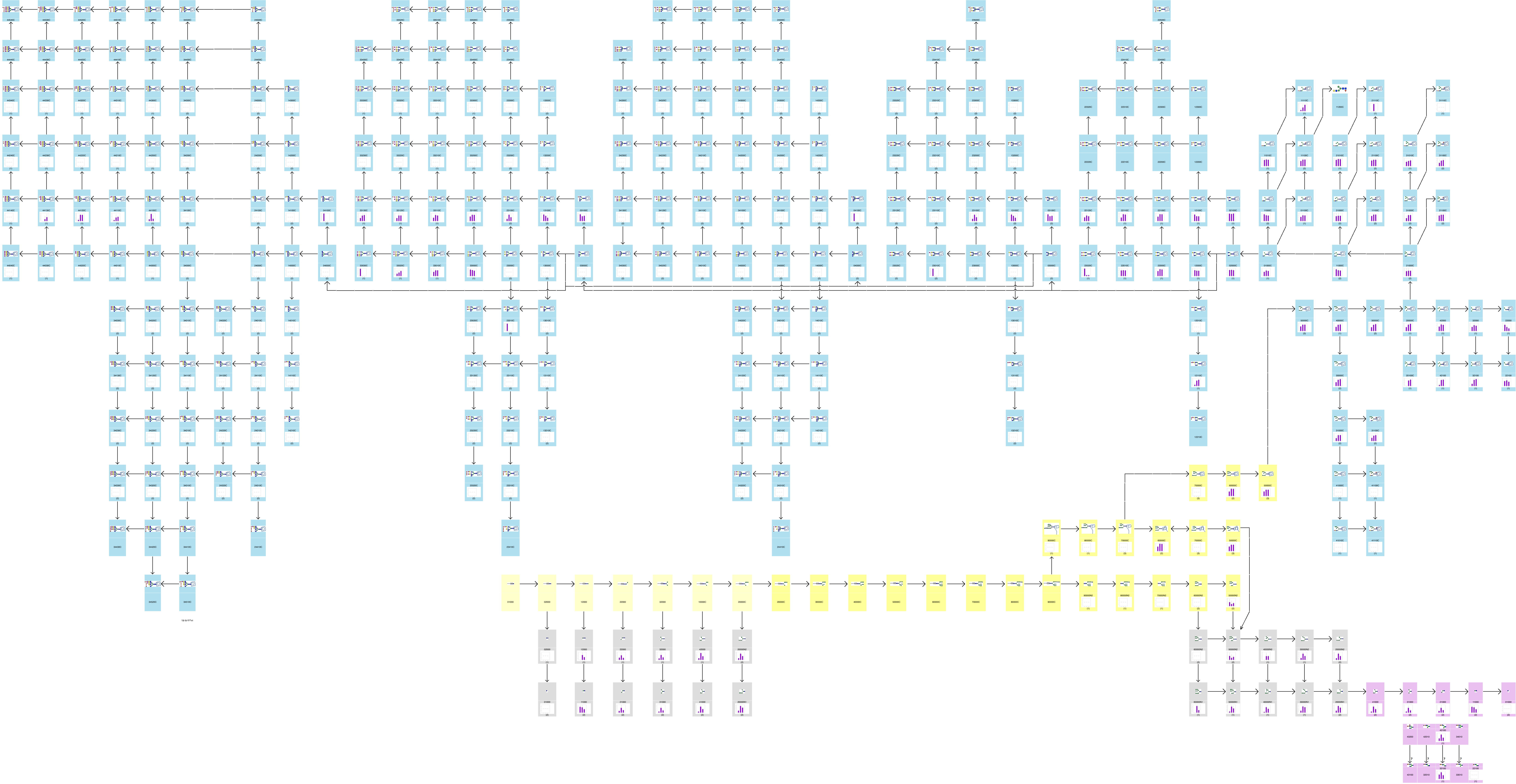

Supplement: Supplementary file 1 [file biomolecules-10-01383-s001.zip › Figure S3.pdf]

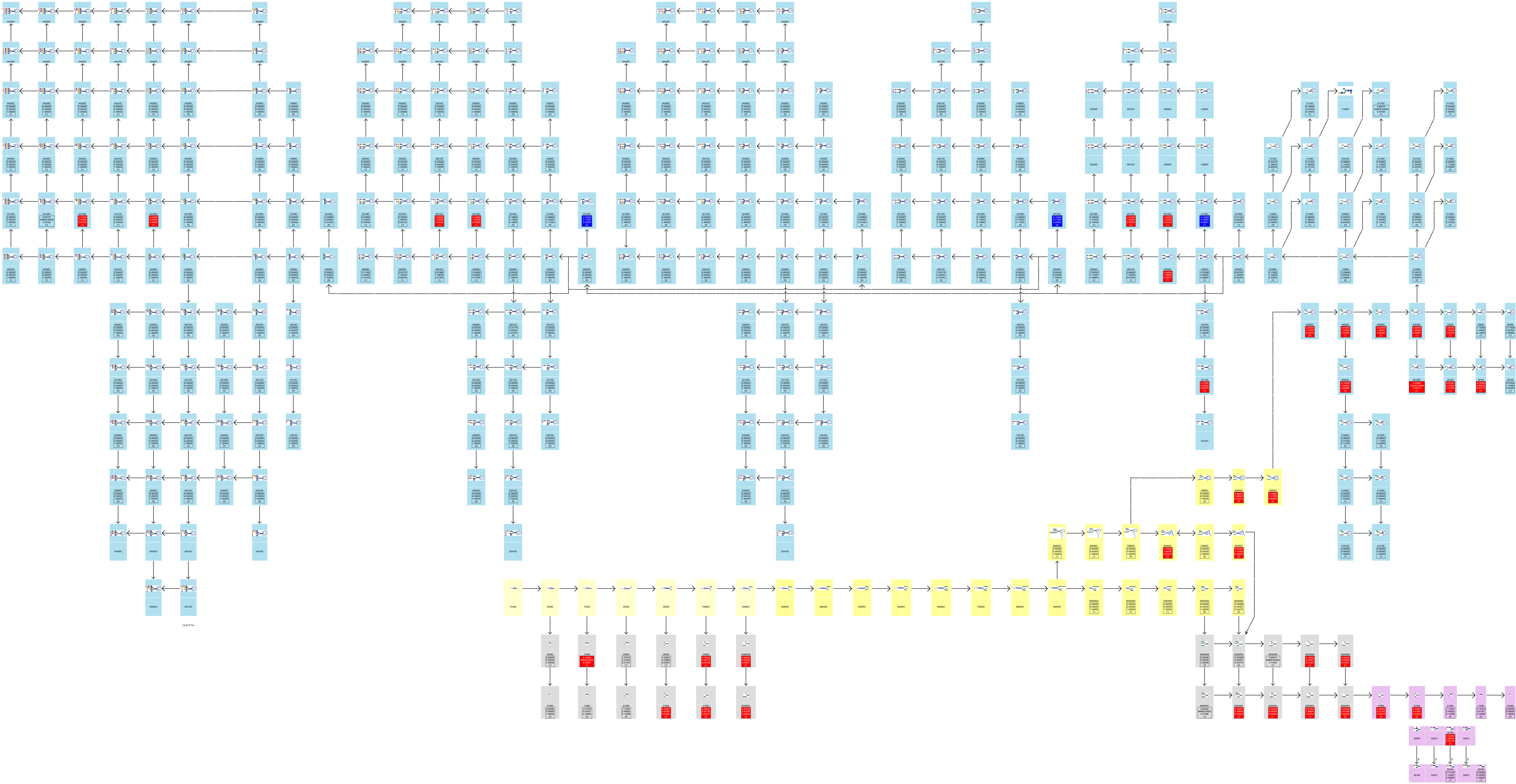

Supplement: Supplementary file 1 [file biomolecules-10-01383-s001.zip › Figure S4.pdf]

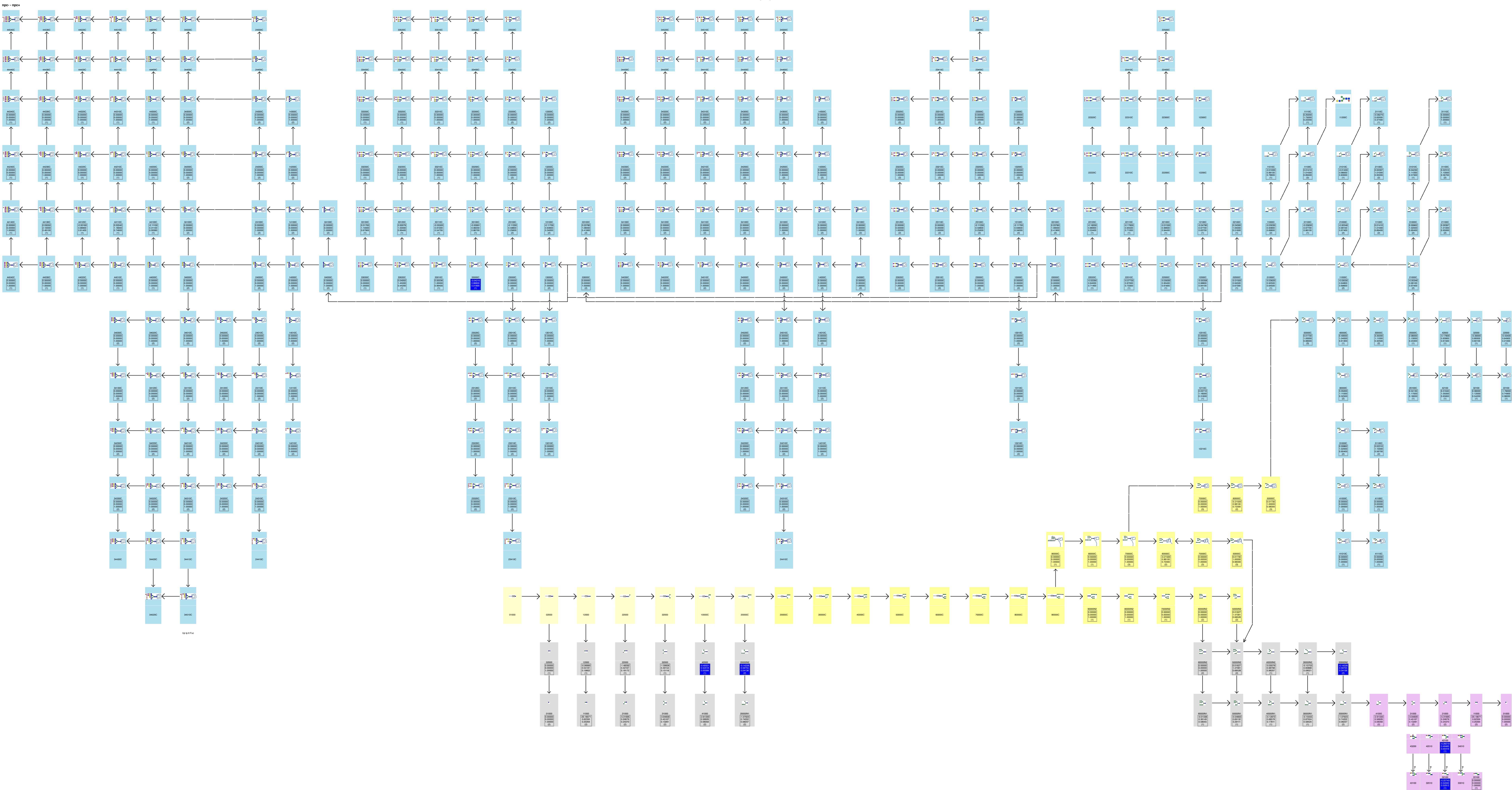

Supplement: Supplementary file 1 [file biomolecules-10-01383-s001.zip › Figure S5.pdf]
